# Supplementary material for: Asynchronous glutamate release is enhanced in low release efficacy synapses and dispersed across the active zone
Source: Nat Commun. 2022 Jun 17;13:3497. doi: 10.1038/s41467-022-31070-4 (PMC9206079; doi:10.1038/s41467-022-31070-4)
Supplement: Supplementary file 1 — Supplementary information [file 41467_2022_31070_MOESM1_ESM.pdf]

Supplementary Information

Title: Asynchronous glutamate release is enhanced in low release efficacy synapses and dispersed across the active zone.

Author list:

Philipe R. F. Mendonça[1,2]*, Erica Tagliatti[1], Helen Langley[1], Dimitrios Kotzadimitriou[1], Criseida G. Zamora-Chimal[3], Yulia Timofeeva[1,3]* and Kirill E. Volynski[1]*

Affiliations:

[1]University College London Institute of Neurology; London, UK;

[2]Department of Physiology and Biophysics, Federal University of Minas Gerais; Brazil;

[3]Department of Computer Science, University of Warwick; Coventry, UK;

*Corresponding authors. k.volynski@ucl.ac.uk, philiperfm@gmail.com and y.timofeeva@warwick.ac.uk

This PDF file includes:

Supplementary Figs. 1 to 15
Supplementary Tables 1 and 2

Other Supplementary Materials for this manuscript include the following:

Supplementary Movies 1 and 2

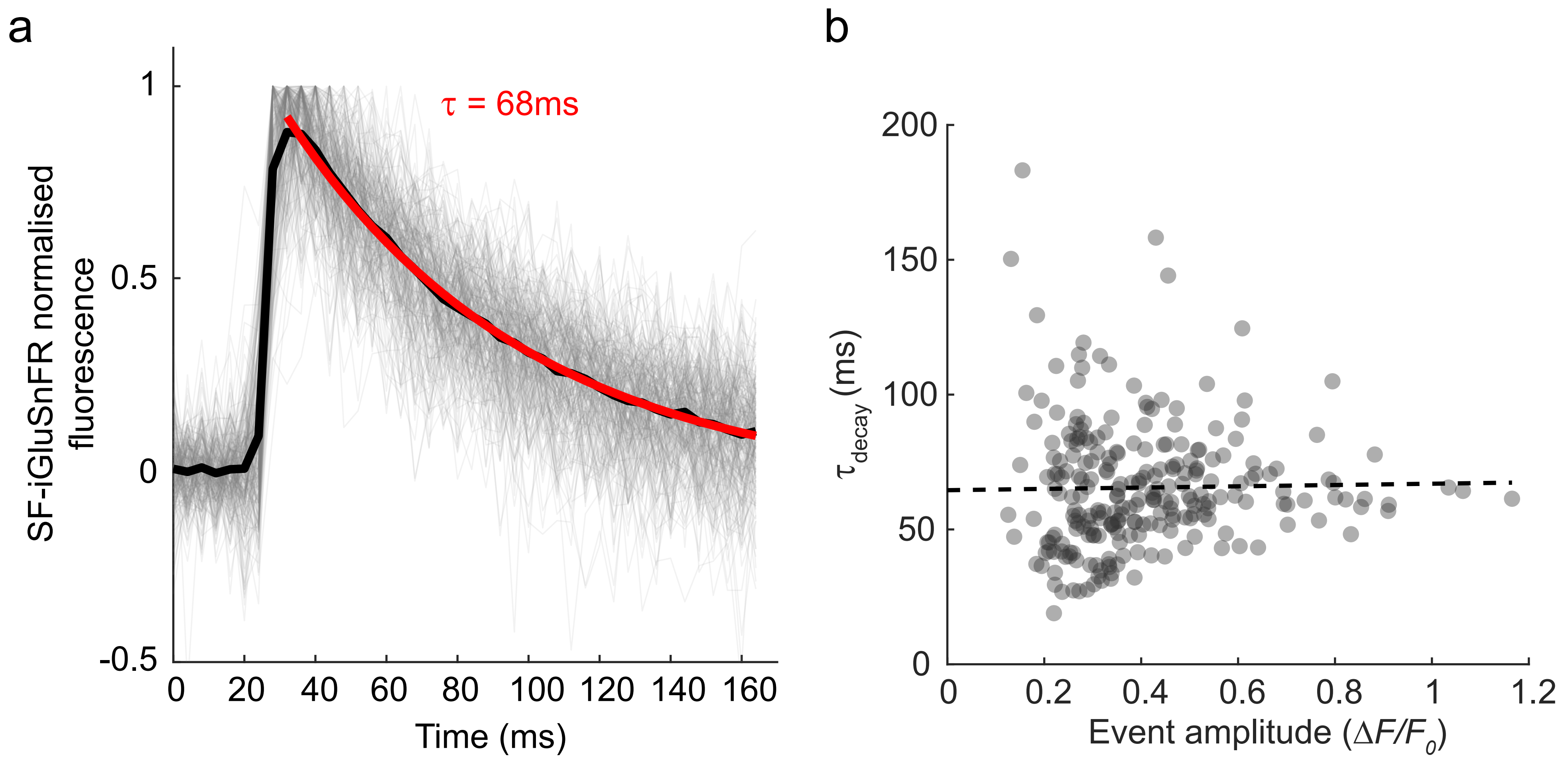

Mendonca et al. Supplementary Fig. 1

Supplementary Fig. 1 Analysis of kinetics of evoked presynaptic SF-iGluSnFR events.

a Black trace, average of 244 individual action potential-evoked SF-iGluSnFR fluorescence transients (grey traces) aligned by their rising slopes (n = 37 representative presynaptic boutons recorded in N = 6 wild type cells). Red trace, mono-exponential fit of the decay phase of the average trace $F(t) = e^{-t/\tau}$.

b The values of decay time constant τ estimated for individual SF-iGluSnFR events do not correlate with the event amplitude. Dashed line, linear regression.

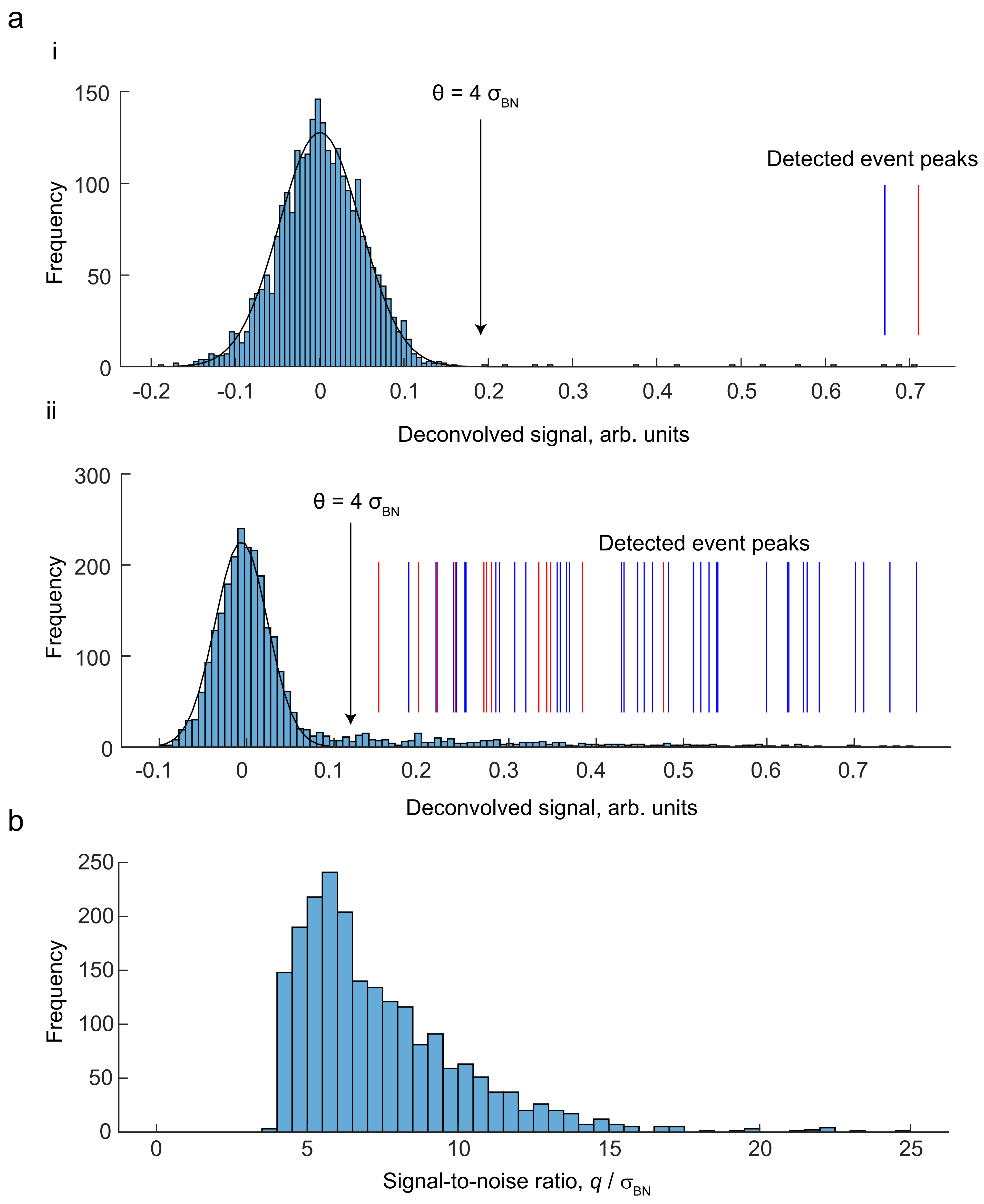

a
i
θ = 4 σBN
Detected event peaks
Frequency
Deconvolved signal, arb. units
ii
θ = 4 σBN
Detected event peaks
Frequency
Deconvolved signal, arb. units
b
Frequency
Signal-to-noise ratio, q / σBN

Supplementary Fig. 2 Analysis of background noise and setting a threshold for automatic detection of quantal glutamate release events in single boutons.
a All-point histograms of the deconvolved traces from boutons 1 and 2 in Fig. 1. Black lines, histogram fits with a Gaussian function $\frac{A}{\sigma_{BN}\sqrt{2\pi}}\exp\left(\frac{-x^2}{2\sigma_{BN}{}^2}\right)$. The obtained standard deviation σ_{BN}, which characterises the baseline noise, was used to set-up the bouton-specific threshold for detection of quantal events: $\theta = 4\,\sigma_{BN}$ (vertical arrows). Vertical bars depict the amplitudes of SF-iGluSnFR synchronous (blue) and asynchronous (red) release events, determined as local maxima located above the threshold on the deconvolved trace in Fig. 1e.
b Distribution of signal-to-noise ratio across recorded synapses (pooled data from N = 16 wild type and N = 11 Syt1$^{-/-}$ neurons, total n = 2,075 boutons). Signal-to-noise ratio in each bouton was calculated as the ratio of SF-iGluSnFR signal amplitude corresponding to release of a single vesicle (1q) to the standard deviation of the background noise σ_{BN}.

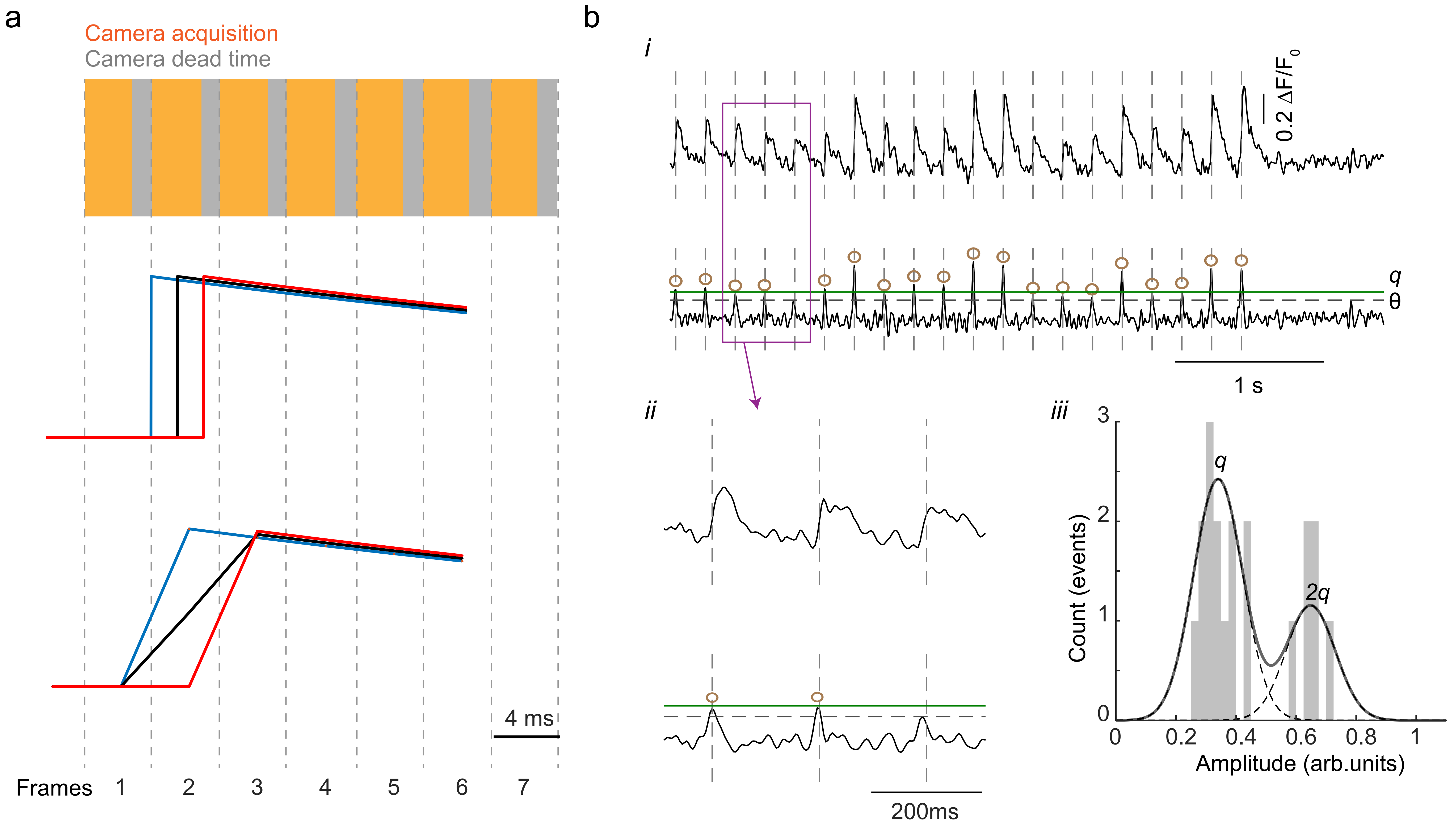
a
Camera acquisition
Camera dead time
4 ms
Frames 1 2 3 4 5 6 7
b
i
0.2 ΔF/F0
q
θ
1 s
ii
200ms
iii
Count (events)
q
2q
Amplitude (arb.units)
0 0.2 0.4 0.6 0.8 1
0 1 2 3

Supplementary Fig. 3 Simulation of SF-iGluSnFR responses mimicking the experimental data.

a Illustration of the predicted effect of a random jitter between the exact vesicular release times and the camera exposure cycle on the profile of SF-iGluSnFR responses. To ensure equal detection of fluorescence signals from all pixels within a selected ROI the Prime95B camera was operated in the 'Pseudo Global Shutter' mode, when excitation light was only delivered during simultaneous exposure of all selected camera rows. As a result, photons were only collected during the first 2.8 ms (yellow rectangles) of the 4 ms frame cycle (vertical dashed lines). Middle traces illustrate 'ideal' SF-iGluSnFR signals that initiate at different times during the frame cycle. Bottom traces depict the shape of corresponding SF-iGluSnFR signals as they would be acquired by the camera.

b Generation and analysis of simulated SF-iGluSnFR responses. Each of 1,000 simulated traces consisted of 1,200 x 4 ms frames. Vesicular release events (20 per trace) were assumed to occur at 5 Hz (*i.e.* every 50th frame). The exact timings of release events within the selected frames were random. The number of vesicles released during each event was drawn from a binomial distribution, assuming $m =$ 5 (the number of release ready vesicles) and $p_v = 0.15$ (release probability of an individual vesicles). The amplitudes of SF-iGluSnFR responses corresponding to simultaneous release of i vesicles were approximated as $\mu_i = \sum_{k=1}^{i} \lambda^{(k-1)} \mu_1$, where $\lambda =$ 0.9 is a factor (estimated experimentally) that accounts for a progressive saturation of SF-iGluSnFR during multi-vesicular release (see Methods). The time course of SF-iGluSnFR responses was approximated as an instantaneous rise followed by an exponential decay with a characteristic time τ = 68 ms (Supplementary Fig. 1). The signal-to-noise ratio for each trace was randomly drawn from the experimentally determined distribution (Supplementary Fig. 2b).

(i) Representative band-pass filtered (top) and deconvolved (bottom) simulated SF-iGluSnFR signals with signal-to-noise ratio 5.7 q / σ_{BN}. As in the case of experimental traces (Fig. 1e), release events (brown circles) were identified as local maxima on the deconvolved trace located above the threshold $\theta = 4\,\sigma_{BN}$ (horizontal dashed line) corresponding to 4 standard deviations of the background noise.

(ii) Enlarged traces corresponding to the boxed area containing a false-negative (missed) event.

(iii) Quantal analysis of the example artificial trace performed as detailed in Fig. 1e.

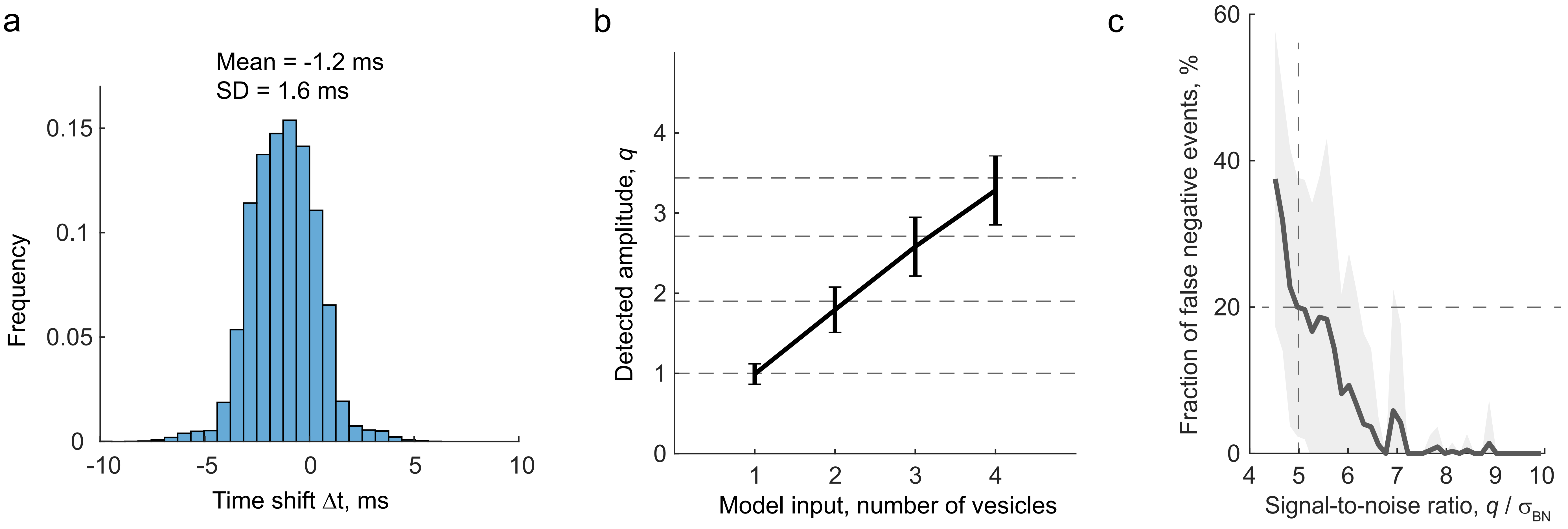

a
Mean = -1.2 ms
SD = 1.6 ms
Frequency
0.15
0.1
0.05
0
-10
-5
0
5
10
Time shift Δt, ms
b
Detected amplitude, q
4
3
2
1
0
1
2
3
4
Model input, number of vesicles
c
Fraction of false negative events, %
60
40
20
0
4
5
6
7
8
9
10
Signal-to-noise ratio, q / σBN

Supplementary Fig. 4 Temporal resolution and sensitivity of quantal analysis.

Simulated traces generated in Supplementary Fig. 3 were used to estimate the temporal resolution and the sensitivity of SF-iGluSnFR quantal analysis (N = 1,000 traces containing 20,000 events, of which 18,813 were detected using the $\theta = 4\sigma_{BN}$ thershold).

a Distribution of the time shifts (Δt) between the determined and the actual (pre-defined) times of simulated events. The results of simulations confirm that temporal resolution of our analysis is limited by the camera acquisition rate (4 ms/frame). The detected event times were systematically shifted to the left by approximately 1.25 ms. This was due to a combined effect of noise, bandpass filtering and the inherent random jitter between the timings of release events and the camera exposure cycle. Considering the intrinsic 2 – 6 ms delay in the onset of synchronous release caused by the finite speed of action potential propagation and variable distances between synapses and cell soma[23] we did not perform correction for this small systematic error.

b Relationship between model input (number of vesicles) and model output (detected amplitude of deconvolved SF-iGluSnFR signal in units of q) verifying the robustness of the amplitude histogram fitting approach. Horizontal dashed lines depict model-predicted SF-iGluSnFR amplitude values ($\mu_i = \sum_{k=1}^{i} \lambda^{(k-1)} \mu_1$, see Methods), mean ± SEM. Numbers of events analysed in each model input group were: 1 vesicle – 12,894; 2 vesicles – 4,932; 3 vesicles – 906; 4 vesicles – 79.

c Dependency of false-negative event fraction on the signal-to-noise ratio. Based on this simulation, boutons with signal-to-noise ratio below $5q/\sigma_{BN}$ (vertical dashed line) were excluded from analysis, thus restricting the fraction of false-negative events to ~20% (horizontal dashed line), mean ± SEM (shaded area).

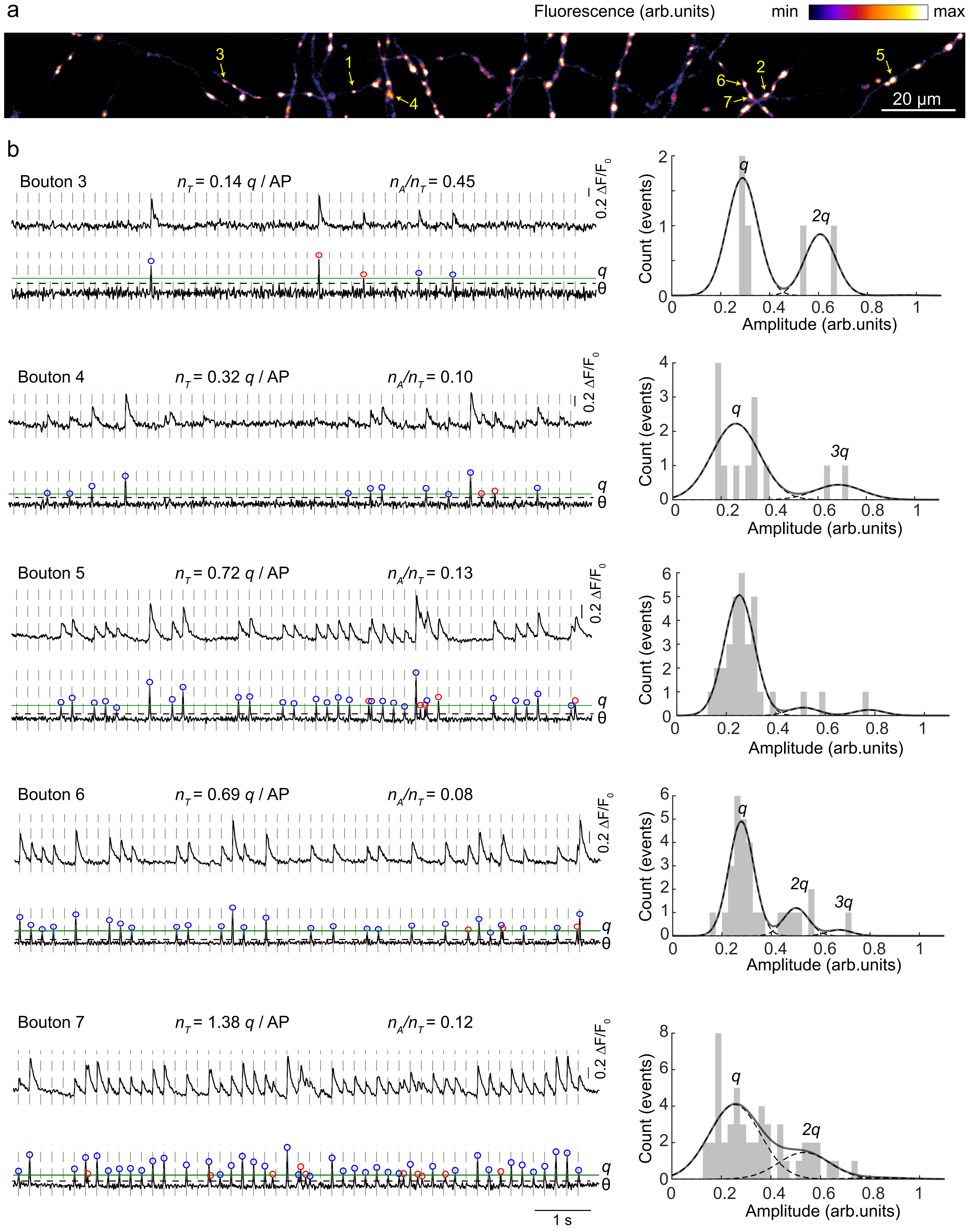
a
Fluorescence (arb.units)
min
max
20 µm
b
Bouton 3
n_T = 0.14 q / AP
n_A/n_T = 0.45
0.2 ΔF/F0
Bouton 4
n_T = 0.32 q / AP
n_A/n_T = 0.10
Bouton 5
n_T = 0.72 q / AP
n_A/n_T = 0.13
Bouton 6
n_T = 0.69 q / AP
n_A/n_T = 0.08
Bouton 7
n_T = 1.38 q / AP
n_A/n_T = 0.12
1 s
Count (events)
Amplitude (arb.units)
q
2q
3q
θ

Supplementary Fig. 5 Additional examples of quantal analysis in individual presynaptic boutons (the same wild type neuron as in Fig. 1).

a Heat map of glutamate release sites (maximal projection of the band pass-filtered SF-iGluSnFR image stack, same image as in Fig. 1d). Yellow arrows depict positions of example boutons.

b Quantal analysis of SF-iGluSnFR responses at Boutons 3 to 7 (see Fig.1e for analysis of boutons 1 and 2). Left, band-pass filtered (top traces) and deconvolved (bottom traces) SF-iGluSnFR signals. Vertical dashed lines depict the timings of somatic action potentials. Horizontal black dashed lines denote the detection thresholds (θ) for vesicular release events, corresponding to 4 standard deviations of the background noise. Horizontal green lines denote the amplitudes of SF-iGluSnFR signals corresponding to release of a single vesicle (single quanta, q) determined by fitting positions of peaks on the amplitude histograms (right). Blue and red circles on the deconvolved traces depict synchronous and asynchronous release events respectively. Comparison of quantal responses at boutons 6 and 7 illustrates that SF-iGluSnFR signals in neighbouring boutons are not cross-contaminated by a possible glutamate spillover.

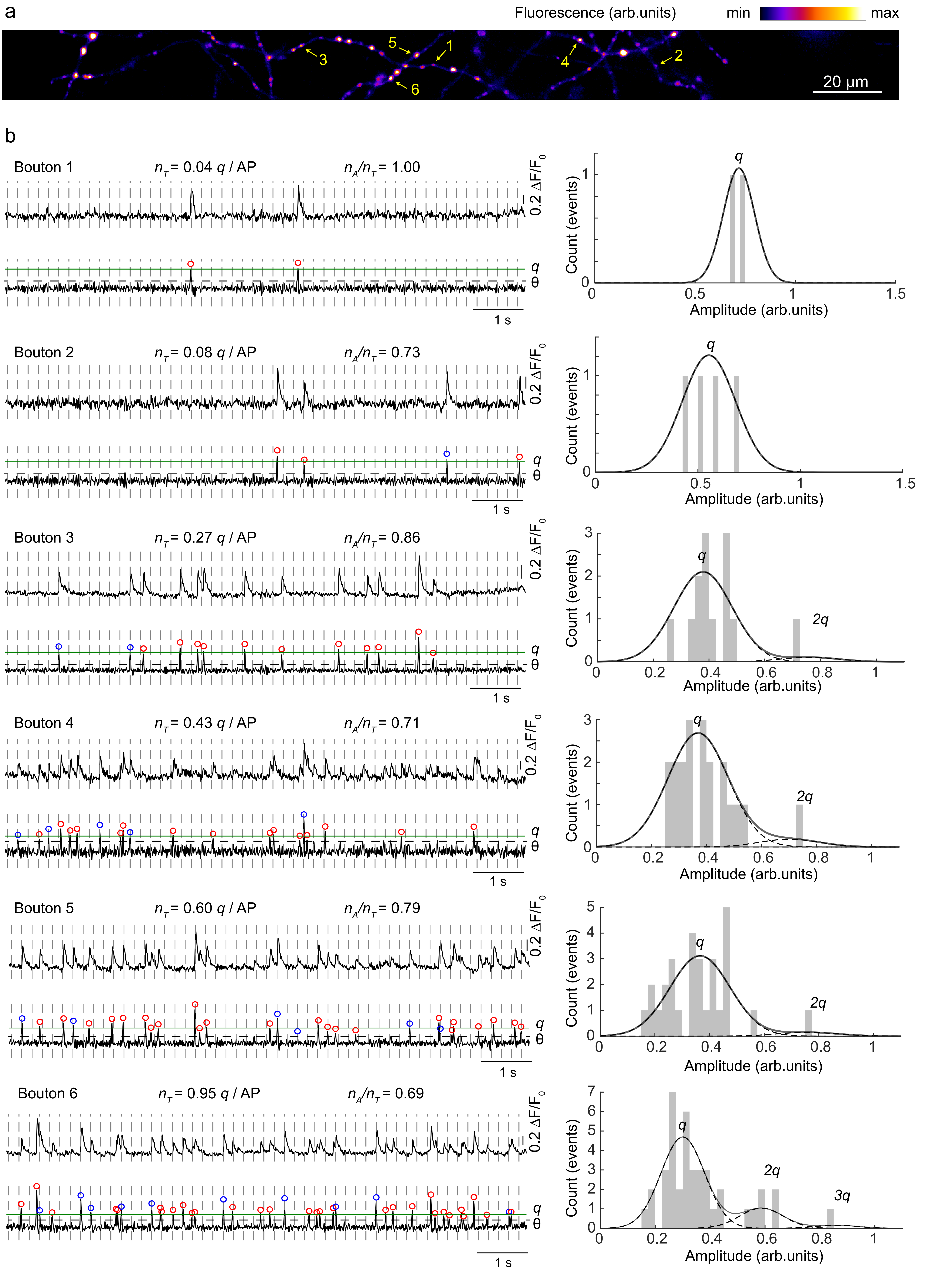

a
Fluorescence (arb.units)
min
max
5
1
3
4
2
6
20 µm
b
Bouton 1
n_T= 0.04 q / AP
n_A/n_T= 1.00
0.2 ΔF/F0
q
θ
1 s
Count (events)
Amplitude (arb.units)
Bouton 2
n_T= 0.08 q / AP
n_A/n_T= 0.73
Bouton 3
n_T= 0.27 q / AP
n_A/n_T= 0.86
2q
Bouton 4
n_T= 0.43 q / AP
n_A/n_T= 0.71
Bouton 5
n_T= 0.60 q / AP
n_A/n_T= 0.79
Bouton 6
n_T= 0.95 q / AP
n_A/n_T= 0.69
3q

Supplementary Fig. 6 Examples of quantal analysis in individual presynaptic boutons in a representative Syt1$^{-/-}$ neuron.

a Heat map of glutamate release sites (maximal projection of the band pass-filtered SF-iGluSnFR image stack) across the axonal arbour of a representative Syt1$^{-/-}$ neuron (total N = 10 Syt1$^{-/-}$ neurons from 4 culture preparations were recorded, see Fig. 2 and Fig. 3b for further analysis). Yellow arrows depict positions of six example boutons.

b Analysis of SF-iGluSnFR responses in the selected boutons. Left, band-pass filtered (top traces) and deconvolved (bottom traces) SF-iGluSnFR signals. Vertical dashed lines depict the timings of somatic action potentials. Horizontal black dashed lines denote the detection thresholds (θ) for vesicular release events, corresponding to 4 standard deviations of the background noise. Horizontal green lines denote the amplitudes of SF-iGluSnFR signals corresponding to release of a single vesicle (q) determined by fitting positions of peaks on the amplitude histograms (right). Blue and red circles on the deconvolved traces depict synchronous and asynchronous release events respectively.

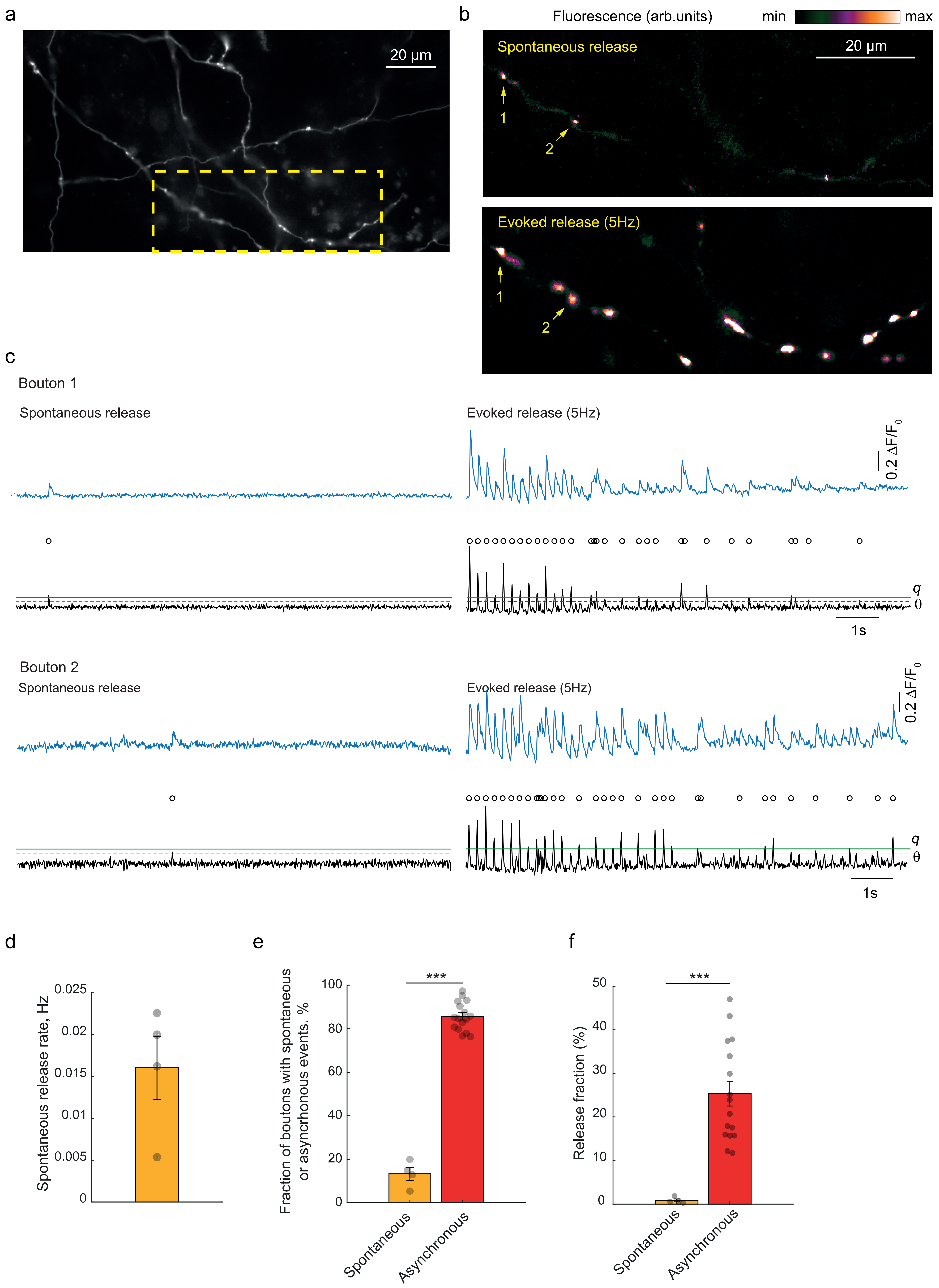

a
20 μm
b
Fluorescence (arb.units)
min
max
Spontaneous release
20 μm
1
2
Evoked release (5Hz)
1
2
c
Bouton 1
Spontaneous release
Evoked release (5Hz)
0.2 ΔF/F0
q
θ
1s
Bouton 2
Spontaneous release
Evoked release (5Hz)
0.2 ΔF/F0
q
θ
1s
d
Spontaneous release rate, Hz
0.025
0.02
0.015
0.01
0.005
0
e
Fraction of boutons with spontaneous or asynchronous events, %
***
100
80
60
40
20
0
Spontaneous
Asynchronous
f
Release fraction (%)
***
50
40
30
20
10
0
Spontaneous
Asynchronous

Supplementary Fig. 7 Estimation of the relative contribution of spontaneous release to the vesicular release recorded during 5 Hz action potential stimulation.

a - **c** A representative experiment (total number of experiments N = 4). (**a**) Example of an axonal arbour of a wild type neuron expressing SF-iGluSnFR. (**b**) Maximal projection of the band-pass filtered image stack (a zoom into the yellow boxed area in (**a**)) revealing locations of all presynaptic boutons that released at least one vesicle either during 10 seconds in the absence of simulation (top, Spontaneous release; the neuron was held at -70 mV) or in response to a 5 Hz train of 51 action potentials (bottom). To increase the imaging area, camera rate in this set of experiments was set to 83.3 Hz (12 ms per frame). (**c**) Analysis of quantal SF-iGluSnFR responses in two boutons that showed spontaneous release (Boutons 1 and 2 in (**b**)). Band-pass filtered (blue) and deconvolved (black) SF-iGluSnFR signals. Quantal release events were identified as local maxima on the deconvolved traces located above the threshold θ (horizontal dashed lines) corresponding to 4 standard deviations of the background noise. The amplitudes of SF-iGluSnFR signals corresponding to release of a single vesicle (q, green lines) were estimated using quantal analysis as described in Fig. 1 and Methods.

d Distribution of the average frequency of spontaneous release (per bouton) among the recorded cells (mean ± SEM).

e Fractions of boutons where spontaneous or asynchronous releases were detected (mean ± SEM).

(**f**) Comparison of average fractions of spontaneous release (in the absence of stimulation) and of asynchronous release during 5 Hz train (mean ± SEM).

(**d- f**) Spontaneous release: N = 4 cells, range 20 – 73 boutons per cell; (**e**, **f**) asynchronous release: data are from wild type cells recorded in Fig. 1; N = 16 cells, range 21 – 162 boutons per cell. *** $p < 0.01$, two-tailed t-test (for further details of statistical analysis see SourceData.xlsx file).

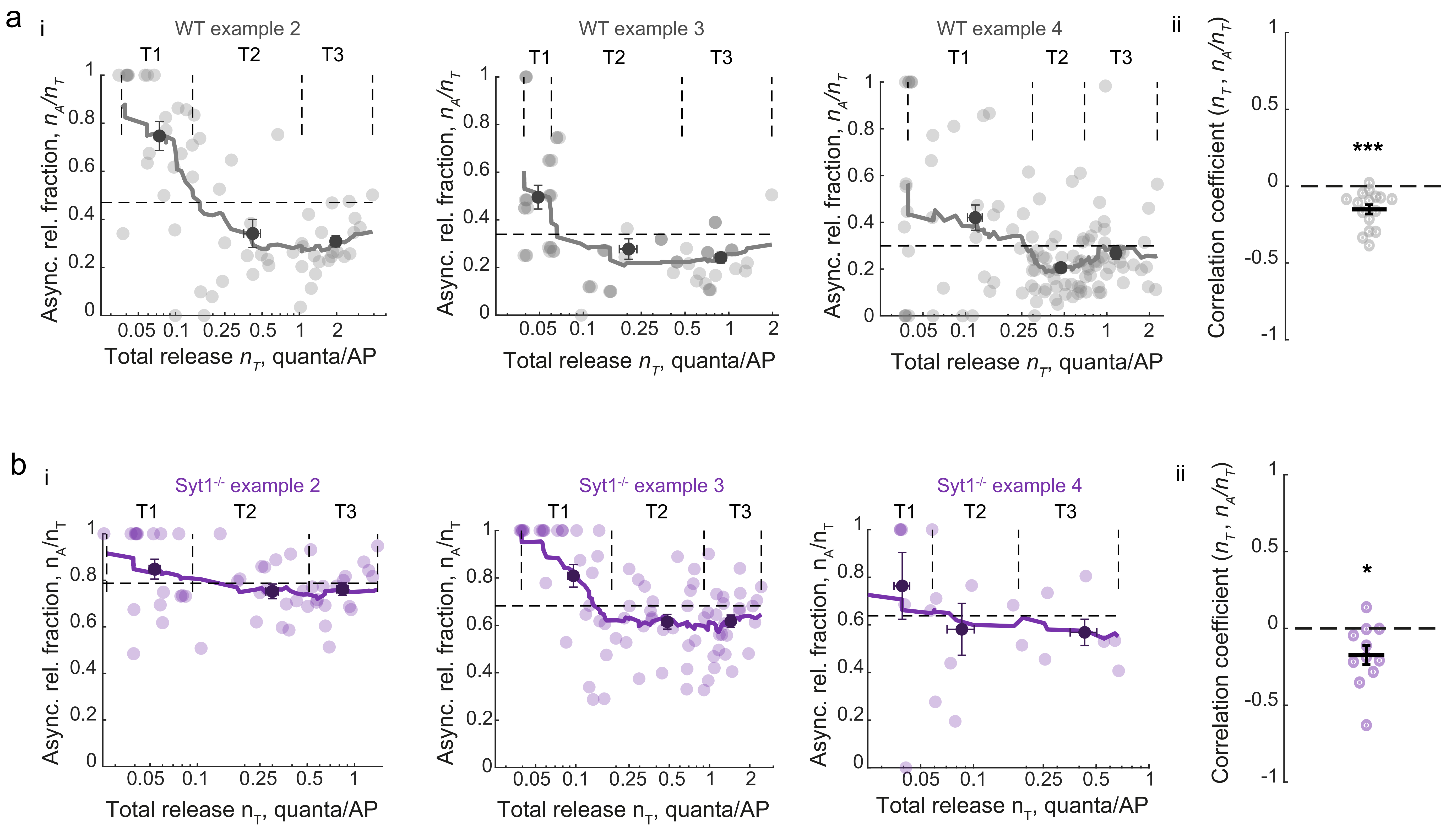

a
i
WT example 2
WT example 3
WT example 4
T1
T2
T3
Async. rel. fraction, n_A/n_T
Total release n_T, quanta/AP
ii
Correlation coefficient (n_T, n_A/n_T)
***
b
Syt1$^{-/-}$ example 2
Syt1$^{-/-}$ example 3
Syt1$^{-/-}$ example 4
Async. rel. fraction, n_A/n_T
Total release n_T, quanta/AP
*

Supplementary Fig. 8 Asynchronous release fraction is increased in synapses with low release efficacy (related to Fig. 3).

(i) Analysis of synchronous and asynchronous release heterogeneity among presynaptic outputs of individual wild type (**a**) and Syt1$^{-/-}$ (**b**) neurons during a 5 Hz train of 51 action potentials. The relationships between asynchronous release fraction n_A/n_T and the overall vesicular release efficacy n_T among boutons in additional representative neurons from wild type (WT example 2, n = 54 boutons; WT example 3, n = 68 boutons; WT example 4, n = 115 boutons) and Syt1$^{-/-}$ (Syt1$^{-/-}$ example 2, n = 51 boutons; Syt1$^{-/-}$ example 3, n = 79 boutons; Syt1$^{-/-}$ example 4, n = 21 boutons) cultures. As in Fig. 3, horizontal dashed lines depict the average fractions of asynchronous release in each cell. Continuous lines, moving averages (20 points span). Vertical dashed lines mark the subdivision of boutons into three terciles with low (T1), intermediate (T2) and high (T3) n_T values. Black dots with error bars depict average values of n_T and n_A/n_T in each tercile (mean ± SEM).

(ii) Distribution of Pearson's correlation coefficients between n_T and n_A/n_T in the recorded cells. Wild type, N = 16 cells from 5 culture preparations; Syt1$^{-/-}$, N = 11 cells from 4 culture preparations. * $p < 0.05$, *** $p < 0.001$, One-sample two-tailed t-test. (for exact p values and for further details of statistical analysis see SourceData.xlsx file)

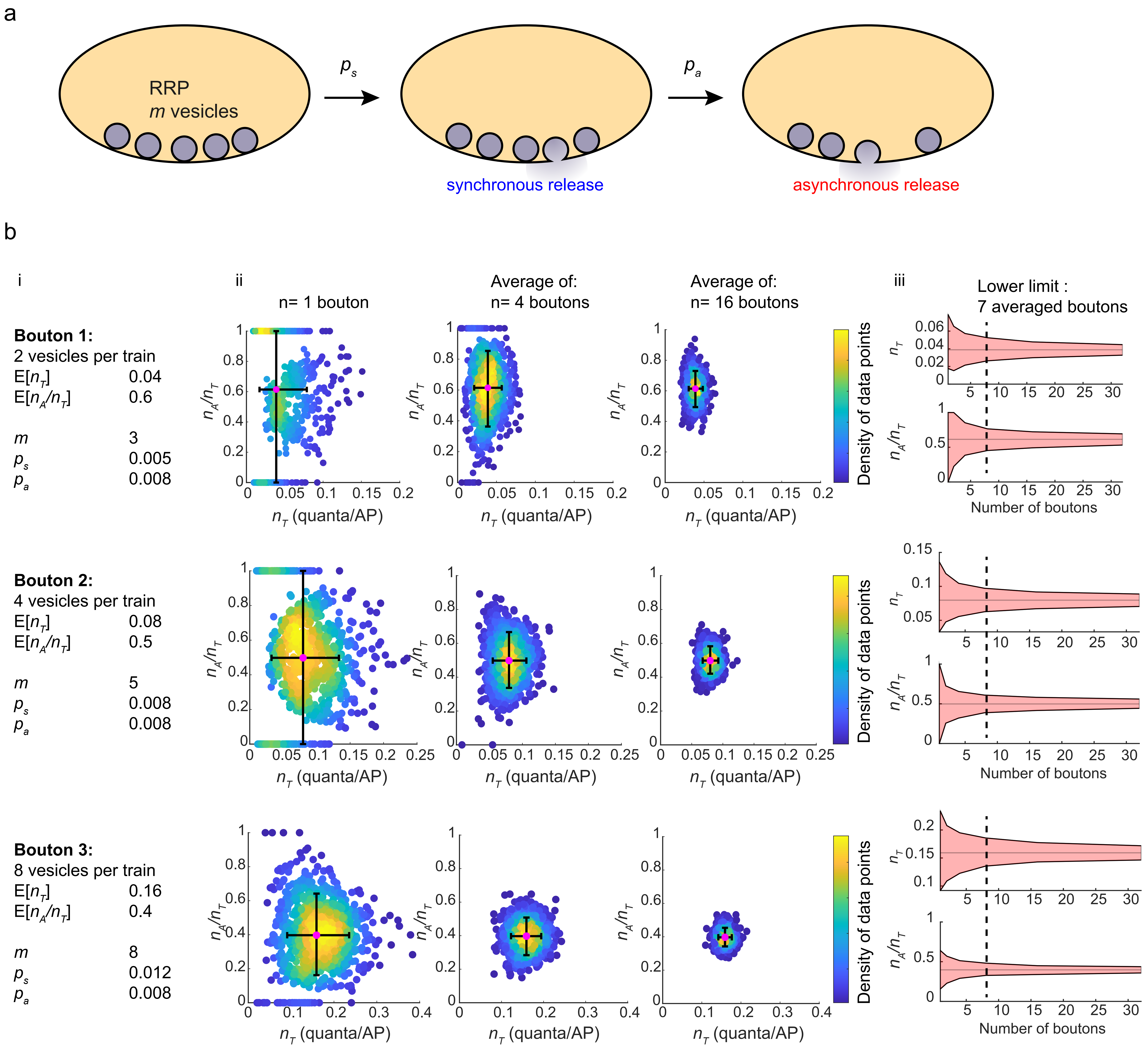
a
p_s
p_a
RRP
m vesicles
synchronous release
asynchronous release
b
i
ii
iii
n= 1 bouton
Average of:
n= 4 boutons
Average of:
n= 16 boutons
Lower limit :
7 averaged boutons
Bouton 1:
2 vesicles per train
$E[n_T]$ 0.04
$E[n_A/n_T]$ 0.6
m 3
p_s 0.005
p_a 0.008
Bouton 2:
4 vesicles per train
$E[n_T]$ 0.08
$E[n_A/n_T]$ 0.5
m 5
p_s 0.008
p_a 0.008
Bouton 3:
8 vesicles per train
$E[n_T]$ 0.16
$E[n_A/n_T]$ 0.4
m 8
p_s 0.012
p_a 0.008
n_A/n_T
n_T (quanta/AP)
Density of data points
n_T
Number of boutons

Supplementary Fig. 9 Evaluation of confidence intervals for measurements of n_T and n_A / n_T in low release efficacy synapses.

a Schematics of Monte Carlo simulations used to model the occurrence of synchronous and asynchronous release events during 5 Hz train of 51 action potentials. We considered a presynaptic bouton containing m RRP vesicles and assumed that each RRP vesicle can undergo synchronous release with probability p_s and asynchronous release with probability p_a. We first simulated whether a given vesicle undergoes synchronous release and, in the case of failure, simulated whether the same vesicle undergoes asynchronous release. By repeating this procedure for all RRP vesicles for each action potential in the train, we thus obtained efficacies of synchronous (n_S) and asynchronous (n_A) release and calculated total release efficacy $n_T = n_S + n_A$ and n_A / n_T fraction. This corresponded to measurements of these parameters during a single imaging sweep in the modelled presynaptic bouton.

For each modelled bouton we also calculated the expected values of n_T and n_A / n_T as follows. According to binomial statistics, the expected value of n_S is $E[n_S] = mp_s$. By considering depletion of RRP during synchronous release phase, we obtain the expected value of asynchronous release efficacy $E[n_A] = (1 - p_s)mp_a$. Then we obtain $E[n_T] = m(p_s + (1 - p_s)p_a)$ and $E[n_A / n_T] = \dfrac{(1 - p_s)p_a}{p_s + (1 - p_s)p_a}$.

b Estimation of confidence intervals in three representative boutons with low release efficacies. (i) We varied the functional presynaptic parameters m, p_s and p_a within physiological range and, using the derived expressions for $E[n_T]$ and $E[n_A / n_T]$, designed three model boutons that would, on average, release 2 (Bouton 1), 4 (Bouton 2) and 8 (Bouton 3) vesicles during 5 Hz train of 51 action potentials. (ii) Left column. For each of the modelled boutons, we performed N = 1,000 simulations and plotted: the obtained n_T and n_A / n_T values (colour depicts relative density of data points: cold colours represent low and warm colours represent high density respectively), 80% confidence intervals for n_T and n_A / n_T (black horizontal and vertical bars), and the expected values of $E[n_A]$ and $E[n_A / n_T]$ (pink dots). To quantify the effect of averaging on the reduction of confidence intervals, we repeated the same type of simulations multiple times (range 2 – 32 repeats) and calculated mean n_T and n_A / n_T values that corresponded to averaging of data points from several boutons of the same type (*e.g.* middle column – an average of 4 boutons, right column – an average of 16 boutons).

(iii) Dependency of confidence intervals for n_T and n_A / n_T estimates on the number of averaged boutons (N = 1000 simulations for each condition). Vertical dashed lines correspond to the lower limit of confidence intervals in our experimental conditions where we averaged at least 7 boutons per group: each recorded cell contained at least 21 boutons that were subdivided into 3 terciles (see Fig. 3). We note that in the case of paired pulse protocol (Fig. 4), we sampled each bouton with 20 (instead of 51) action potentials. To stay within the same confidence intervals, we averaged at least 17 boutons per group. Therefore, in this set of experiments each recorded cell contained at least 50 boutons.

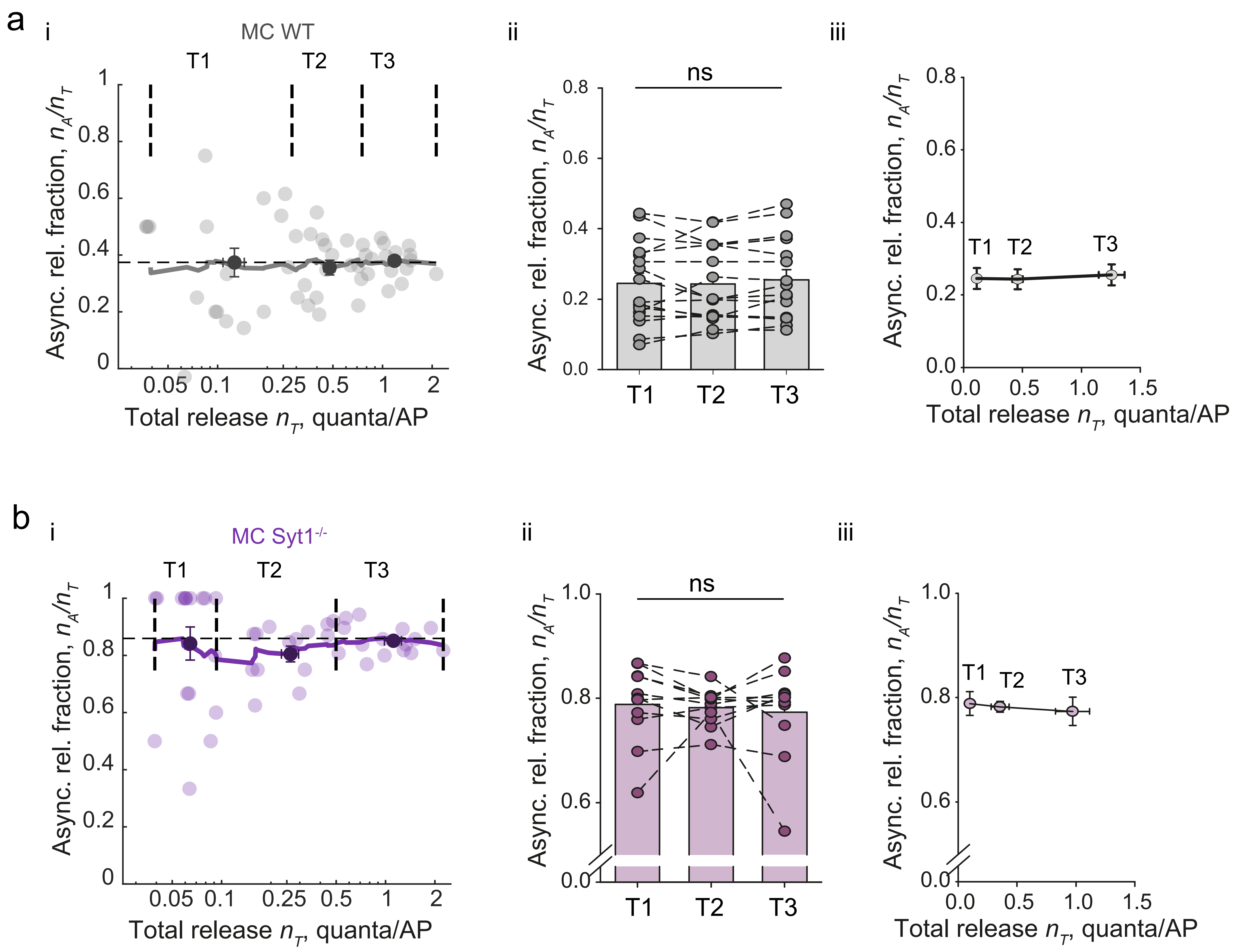
a
i
MC WT
T1
T2
T3
Async. rel. fraction, n_A/n_T
Total release n_T, quanta/AP
ii
ns
T1
T2
T3
iii
b
MC Syt1$^{-/-}$

Supplementary Fig. 10. Control Monte-Carlo simulations related to Fig. 3

As a control, we randomly reassigned the type of each recorded release event (synchronous or asynchronous) in each bouton independently of n_T using the average n_A / n_T value in a given cell and processed the simulated data set using the same analysis routine as for the experimental data set in Fig. 3 (N = 16 wild type and N = 11 Syt1$^{-/-}$ neurons). Our rationale was that if there was any spurious correlation between n_A / n_T and n_T introduced by the data processing, it should also be observed in the analysis of the simulated data. However, the experimentally observed increase of asynchronous release fraction in synapses with low n_T was lost in the control simulations, which argues that this phenomenon was not simply a consequence of a spurious correlation: (i) Monte-Carlo simulations corresponding to the example cells shown in Fig. 3, (ii) corresponding bar and dot plots (mean ± SEM) and (iii) dependences of n_A/n_T versus n_T averaged among all recorded cells (mean ± SEM) for the simulated data; ns $p > 0.5$, repeated measures ANOVA (for exact p values see SourceData.xlsx file).

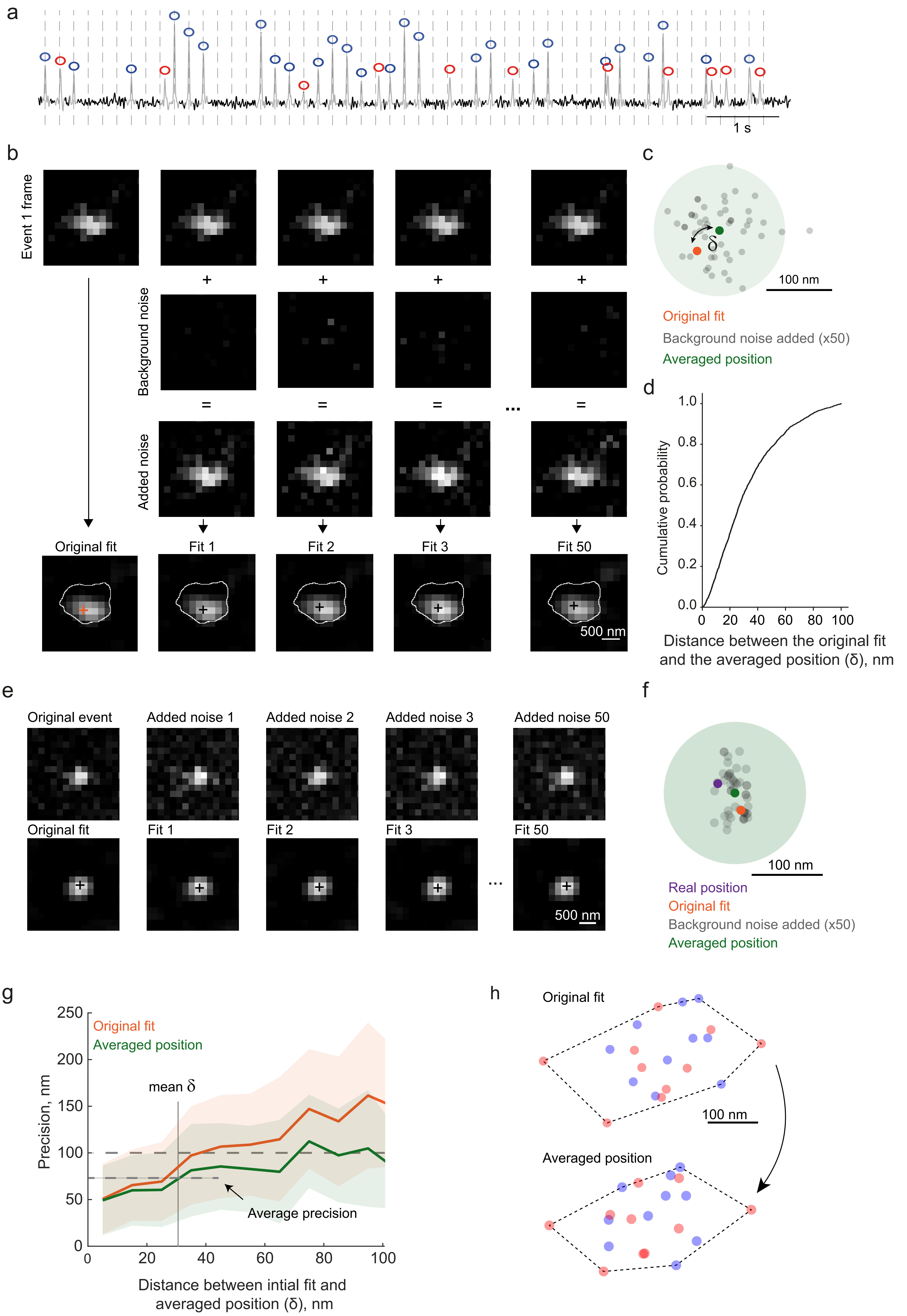
a
1 s
b
Event 1 frame
Background noise
Added noise
Original fit
Fit 1
Fit 2
Fit 3
Fit 50
500 nm
c
δ
100 nm
Original fit
Background noise added (x50)
Averaged position
d
Cumulative probability
Distance between the original fit and the averaged position (δ), nm
e
Original event
Added noise 1
Added noise 2
Added noise 3
Added noise 50
Original fit
Fit 1
Fit 2
Fit 3
Fit 50
500 nm
f
100 nm
Real position
Original fit
Background noise added (x50)
Averaged position
g
Original fit
Averaged position
mean δ
Average precision
Precision, nm
Distance between intial fit and averaged position (δ), nm
h
Original fit
100 nm
Averaged position

Supplementary Fig. 11. Estimation and improvement of vesicular release sites localisation precision using noise analysis.

a Generation of 'background noise' image stack (1000 images) using the information on timing of the release events in the deconvolved SF-iGluSnFR trace. Each background noise image was obtained by averaging 3 randomly selected frames from the deconvolved SF-iGluSnFR image stack, which were separated from the nearest release event by at least 15 frames (depicted in black on the representative trace, the same bouton as in Fig. 5a-d).

b, c Estimation of the precision of vesicular release localisation (detailed illustration for Event 1 from Fig. 5a-d). (**b**) For each event, an Added noise image stack was generated (50 images), where each image was a sum of the original Event image and a randomly selected image from the Background Noise stack. We next ran the sub-pixel localisation analysis for each image in the Added noise stack (grey dots in **c**), calculated their average position (green dot) and the distance (δ) between the location of the Original fit (obtained using the initial Event image, orange dot) and the average position from the Added noise stack. Events with δ above the 100 nm threshold were excluded from the analysis.

d Cumulative distribution of δ values for 2,592 fitted events from n = 106 wild type boutons, δ_{mean} = 32.4 ± 23.0 nm (mean ± standard deviation).

e-g Testing if δ represents a realistic estimate of the localisation accuracy by performing localisation analysis on artificial computer-simulated images (see Methods). (**e**) and (**f**) A representative simulation. The purple point in (**f**) depicts the Real position of the simulated event. (**g**) Dependency of the localisation precision (defined as the distance to the real position either from the Original fit or from the Averaged position) on δ (N = 1500 simulations, shaded areas depict standard deviation). The simulations reveal that the averaged position determined from the Added noise stack provides a more reliable estimate for the location of the release site. Therefore, we used this improved averaged fit in the analysis of the relative distributions of synchronous and asynchronous release events. Considering that δ_{mean} = 32.4, the average accuracy in the localisation of exocytosis events in our experiments was ~ 75 nm (in the range between 50 – 100 nm).

h Comparison of the original and the improved localisation maps for the bouton shown in Fig. 5a-d.

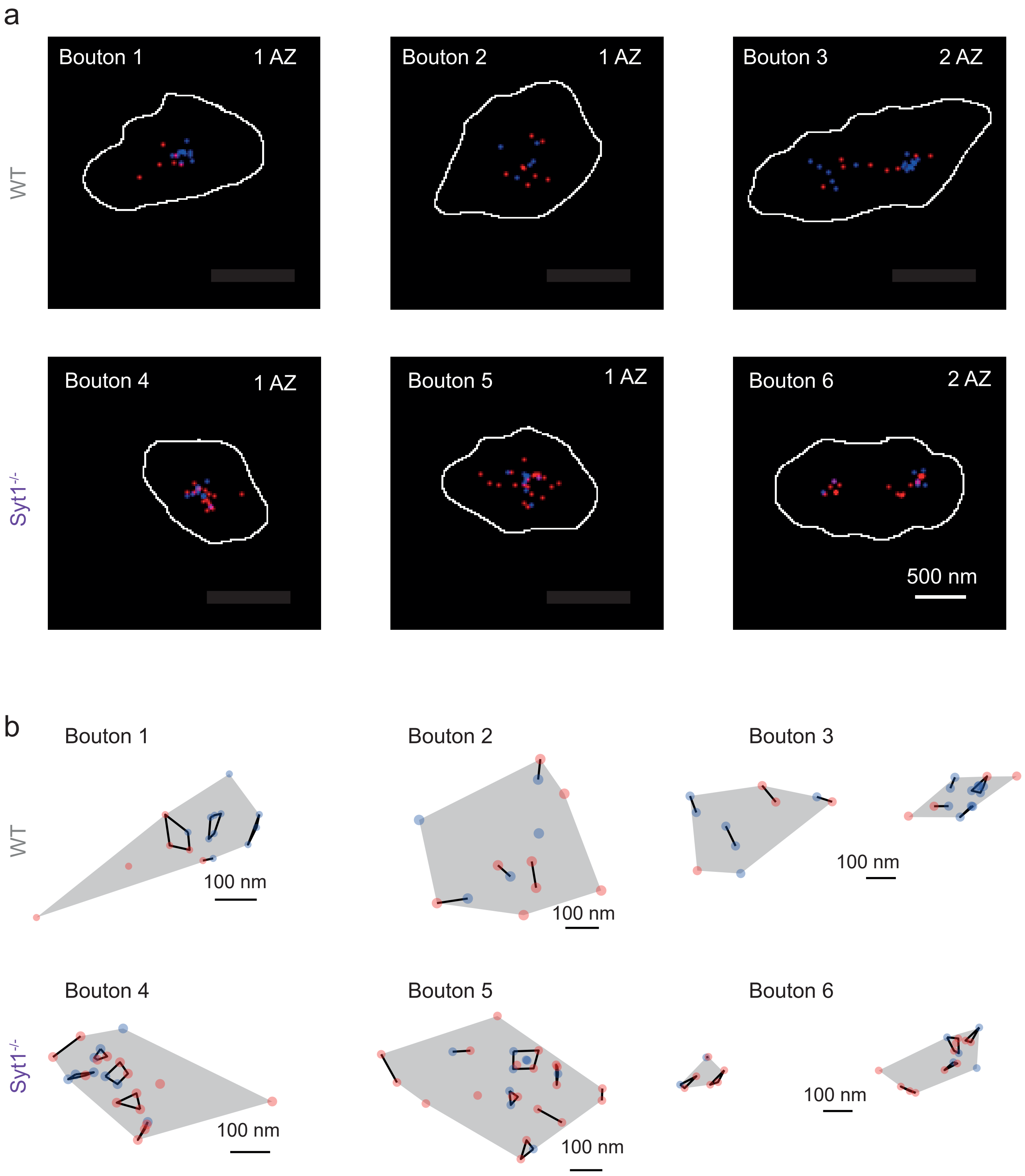
a
Bouton 1
1 AZ
Bouton 2
1 AZ
Bouton 3
2 AZ
WT
Bouton 4
1 AZ
Bouton 5
1 AZ
Bouton 6
2 AZ
Syt1-/-
500 nm
b
Bouton 1
WT
100 nm
Bouton 2
100 nm
Bouton 3
100 nm
Bouton 4
Syt1-/-
100 nm
Bouton 5
100 nm
Bouton 6
100 nm

Supplementary Fig. 12. Additional examples of spatial distributions of synchronous and asynchronous exocytosis sites.

a Composite images showing relative locations of synchronous (blue) and asynchronous (red) release events in several representative wild type boutons (WT, 1-3) and Syt1$^{-/-}$ boutons (4 - 6), see also Fig. 5d.

b Hierarchical cluster analysis of vesicular exocytosis sites in the same boutons as in (**a**). Putative active zones (grey shaded areas) were identified using a clustering diameter threshold of 700 nm. Boutons with 2 active zones (*e.g.* boutons 3 and 6) were excluded from further analysis. To compare the relative locations of synchronous and asynchronous release sites, the positions of vesicular release events were grouped into clusters using a clustering diameter threshold of 100 nm (see Fig. 6a and the main text for details).

In total n = 106 boutons from N=16 cells for wild type and n = 64 boutons from N=11 cells for Syt1$^{-/-}$ neurons passed the selection criteria for sub-pixel localisation (Methods, Data Inclusion and Exclusion Criteria).

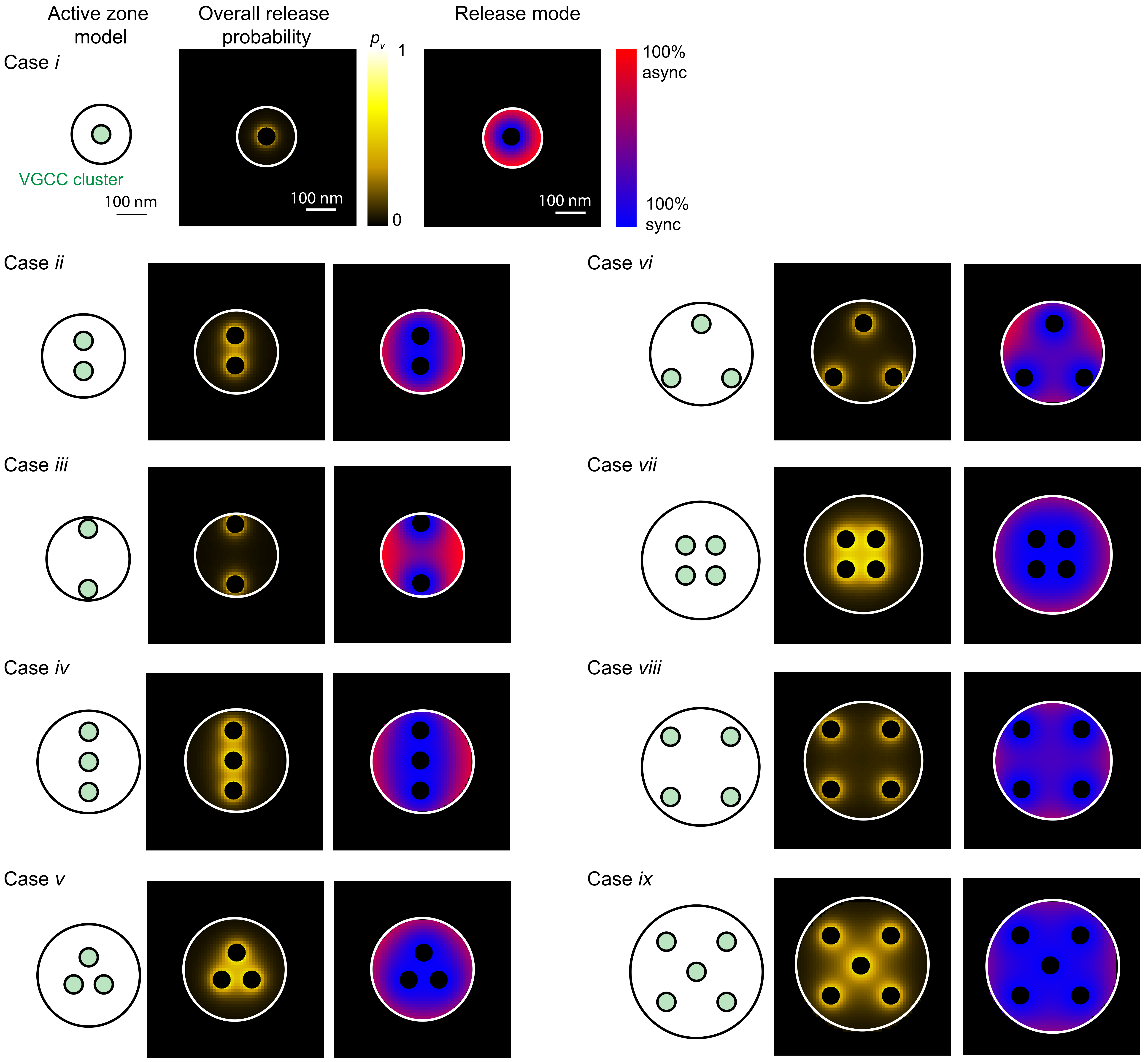

Active zone model
Overall release probability
Release mode
Case i
VGCC cluster
100 nm
100 nm
100 nm
p_v
1
0
100% async
100% sync
Case ii
Case iii
Case iv
Case v
Case vi
Case vii
Case viii
Case ix

Supplementary Fig. 13 related to Fig. 7. Vesicular release probability maps for different active zone geometries for the model with exogenous intracellular pipette buffer.

Nine cases of considered active zone geometries (*i* to *ix*) and corresponding model-predicted maps of the overall release probability $p_v = p_s + (1 - p_s) p_a$, and of the relative fractions of synchronous (blue) and asynchronous (red) release modes for the model with intracellular pipette buffer (see related Fig. 7 and Methods for details).

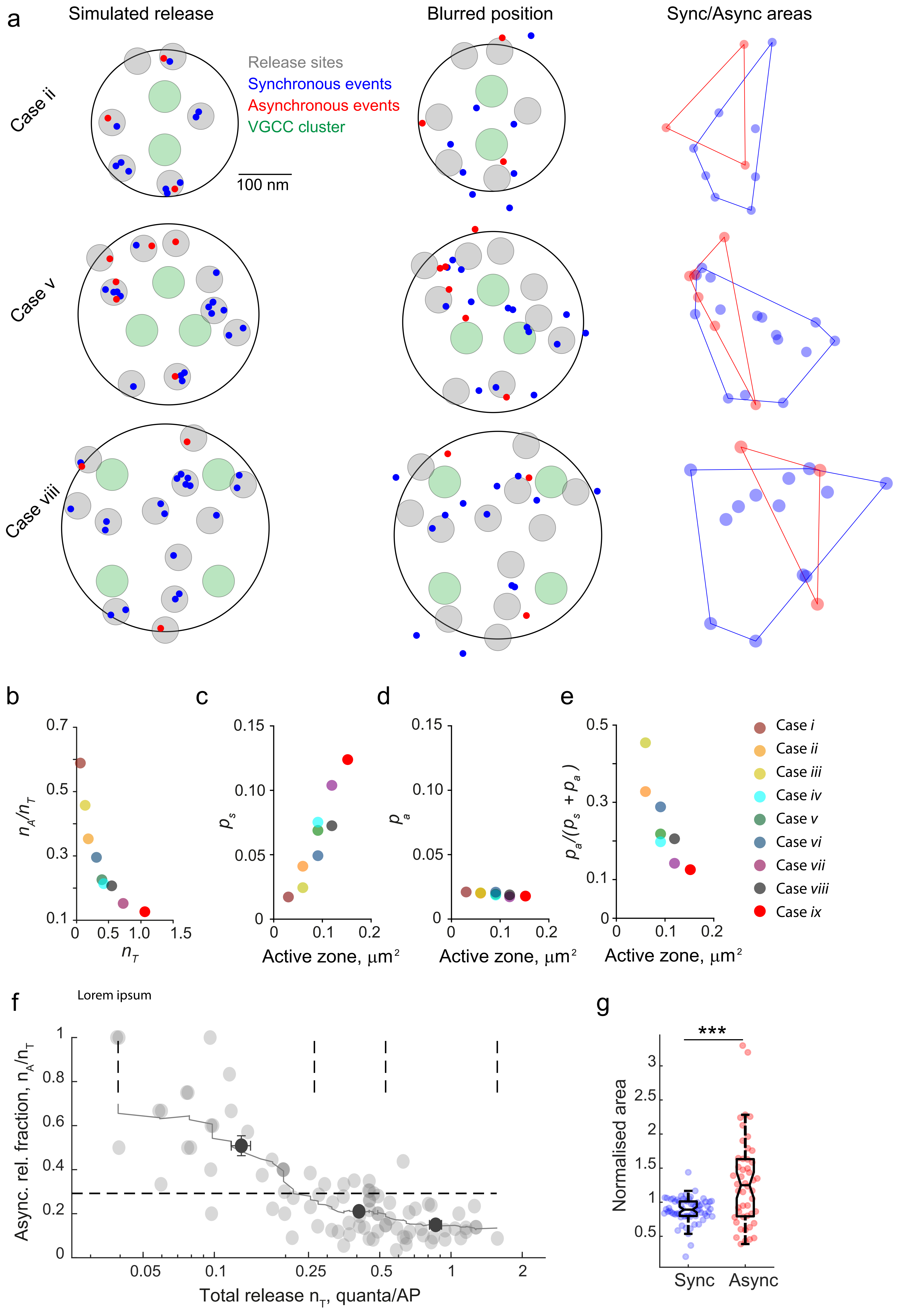

a
Simulated release
Blurred position
Sync/Async areas
Release sites
Synchronous events
Asynchronous events
VGCC cluster
100 nm
Case ii
Case v
Case viii
b
c
d
e
Active zone, µm²
Case i
Case ii
Case iii
Case iv
Case v
Case vi
Case vii
Case viii
Case ix
f
Lorem ipsum
Async. rel. fraction, n_A/n_T
Total release n_T, quanta/AP
g
Normalised area
***
Sync
Async

Supplementary Fig. 14 related to Fig. 8. Simulations of vesicular release in synapses with different active zone geometries (model with exogenous intracellular pipette buffer).

a Results of three representative simulations for active zone geometry cases *ii*, *v* and *viii*. Left, Simulated release. Grey circles depict randomly assigned locations of vesicular release sites. Blue and red dots correspond to synchronous and asynchronous release events respectively, which occurred during a simulated train of 51 action potentials. Middle, Blurred position. To mimic the experimental data, multi-vesicular release events were excluded and locations of the remaining events were blurred by adding a random value corresponding to the accuracy of SF-iGluSnFR event localisation (75 nm). Right, Sync/async areas. Comparison of convex hull areas circumventing simulated blurred locations of synchronous or asynchronous events.

b – **e** Relationships between computed functional presynaptic parameters for nine modelled active zone geometries (averages of n = 250 simulations in each case): dependency of the asynchronous release fraction n_A / n_T on the overall release efficacy n_T (**b**) and dependencies of the probability of synchronous release p_s (**c**), of asynchronous release p_a (**d**) and of model-predicted asynchronous release fraction $p_a /(p_s + p_a)$ on active zone size, calculated for single RRP vesicles. Colour codes are shown on the right. **f**, **g** Comparison of the model's output to the experimental results. Combined outputs from all nine considered geometry cases (n = 10 simulations per case) were processed using the same analysis routine as for the experimental data set. (**f**) Model-predicted relationship between asynchronous release fraction and the overall vesicular release efficacy n_T. Similar to analysis in Fig. 3a horizontal dashed lines depict the average fraction of asynchronous release across all simulated boutons, continuous line – moving average, black dots – average values of n_T and n_A/n_T in each tercile (mean ± SEM). (**g**) Results of reshuffling analysis (similar to Fig. 6d). Data points and box-and-whisker plots: centre, median; notch 95% confidence interval for median; box, 25th – 75th percentiles; whiskers, the most extreme data points not considered outliers (within 1.5 times the interquartile range from the bottom or the top of the box). *** $p < 0.001$, two-tailed Mann–Whitney U test (for exact p values see SourceData.xlsx file).

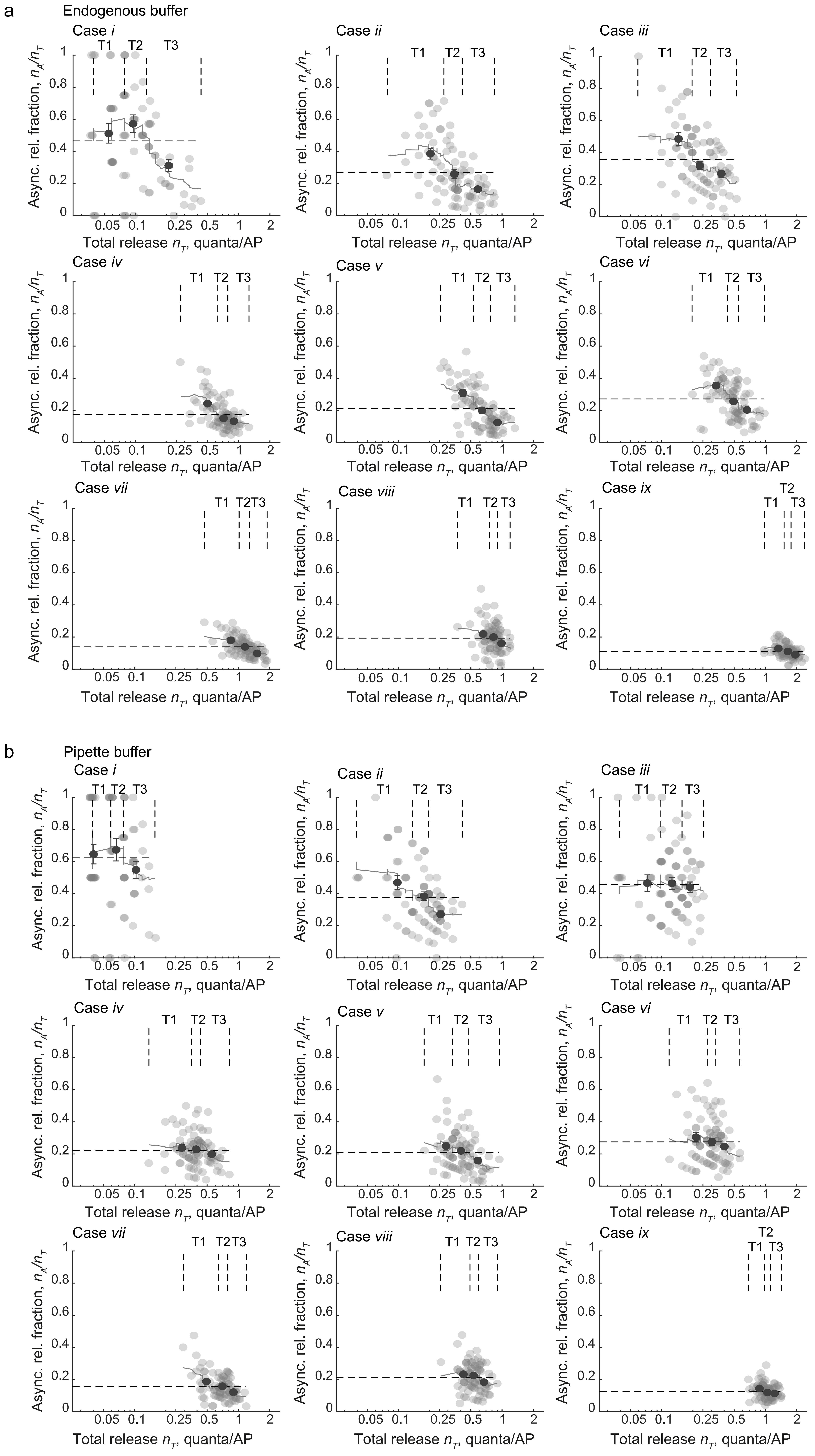

Mendonca et al. Supplementary Fig. 15

Supplementary Fig. 15. Relationships between n_A / n_T and n_T for each considered active zone geometry.

a Model with endogenous presynaptic buffers and **b** model with the intracellular pipette buffer. Outputs from each geometry (n = 90 simulations per case) were processed using the same analysis routine as for the experimental data set in Fig. 3a. Horizontal dashed lines depict the average fractions of asynchronous release for each geometry case. Continuous lines, moving averages (20 points span). Vertical dashed lines mark the subdivision of simulated boutons into three terciles with low (T1), intermediate (T2) and high (T3) n_T values. Black dots with error bars depict average values of n_T and n_A/n_T in each tercile (mean ± SEM).

Supplementary Table 1

Properties of endogenous Ca^{2+} buffers used in VCell model of action-potential evoked Ca^{2+} dynamics.

Calbindin-D28K (refs.[29, 56-58])

Reaction:

$$CB_{fast} + Ca^{2+} \underset{k_{off}^{CB\_fast}}{\overset{k_{on}^{CB\_fast}}{\rightleftarrows}} CaCB_{fast}$$

$$CB_{slow} + Ca^{2+} \underset{k_{off}^{CB\_slow}}{\overset{k_{on}^{CB\_slow}}{\rightleftarrows}} CaCB_{slow}$$

Reaction rates:

$k_{on}^{CB\_fast}$	87 μM^{-1} s^{-1}
$k_{off}^{CB\_fast}$	35.8 s^{-1}
$[CB_{fast}]_{total}$	95 µM
$k_{on}^{CB\_slow}$	11 μM^{-1} s^{-1}
$k_{off}^{CB\_slow}$	2.6 s^{-1}
$[CB_{slow}]_{total}$	95 µM
D_{CB}	20 μm^2 s^{-1}

ATP (refs.[58-60])

Reaction:

$$ATP + Ca^{2+} \underset{k_{off}^{ATP}}{\overset{k_{on}^{ATP}}{\rightleftarrows}} CaATP$$

Reaction rates:

k_{on}^{ATP}	500 μM^{-1} s^{-1}
k_{off}^{ATP}	1.0 x 10^5 s^{-1}
$[ATP]_{total}$	0.9 mM (corresponding to 58 µM $[ATP]_{free}$ at 1 mM $[Mg^{2+}]_{free}$ [60])
D_{ATP}	220 μm^2 s^{-1}

Calmodulin (refs.[60, 61])

Reaction:

N-lobe

$$N_T N_T + Ca^{2+} \underset{k_{off}^{(T),N}}{\overset{2 \cdot k_{on}^{(T),N}}{\rightleftarrows}} CaN_T N_R + Ca^{2+} \underset{2 \cdot k_{off}^{(R),N}}{\overset{k_{on}^{(R),N}}{\rightleftarrows}} CaN_R CaN_R$$

C-lobe

$$C_T C_T + Ca^{2+} \underset{k_{off}^{(T),C}}{\overset{2 \cdot k_{on}^{(T),C}}{\rightleftarrows}} CaC_T C_R + Ca^{2+} \underset{2 \cdot k_{off}^{(R),C}}{\overset{k_{on}^{(R),C}}{\rightleftarrows}} CaC_R CaC_R$$

Reaction rates:

N-lobe

$k_{on}^{(T),N}$	770 μM^{-1} s^{-1}
$k_{off}^{(T),N}$	1.6 x 10^5 s^{-1}
$k_{on}^{(R),N}$	3.2 x 10^4 μM^{-1} s^{-1}
$k_{off}^{(R),N}$	2.2 x 10^4 s^{-1}

C-lobe	
$k_{on}^{(T),C}$	84 μM^{-1} s^{-1}
$k_{off}^{(T),C}$	2.6 x 10^{3} s^{-1}
$k_{on}^{(R),C}$	25 μM^{-1} s^{-1}
$k_{off}^{(R),C}$	6.5 μM^{-1} s^{-1}
$[CaM]_{total}$	100 μM
D_{CaM}	20 μm^{2} s^{-1}

Supplementary Table 2

Properties of exogenous Ca^{2+} buffers (intracellular pipette solution) used in VCell model of action-potential evoked Ca^{2+} dynamics.

**ATP** (refs.[58-60])	
Reaction:	
$ATP + Ca^{2+} \underset{k_{off}^{ATP}}{\overset{k_{on}^{ATP}}{\rightleftarrows}} CaATP$	
Reaction rates:	
k_{on}^{ATP}	500 μM^{-1} s^{-1}
k_{off}^{ATP}	1.0 x 10^5 s^{-1}
$[ATP]_{total}$	4 mM (corresponding to 98 µM $[ATP]_{free}$ at 4 mM $[Mg^{2+}]_{free}$ [60])
D_{ATP}	220 μm^2 s^{-1}
**Gluconate**	
Reaction:	
$Glu^{-} + Ca^{2+} \underset{k_{off}^{Glu}}{\overset{k_{on}^{Glu}}{\rightleftarrows}} CaGlu^{+}$ *	
Reaction rates:	
k_{on}^{Glu}	500 μM^{-1} s^{-1}
k_{off}^{Glu}	2.85 x 10^7 s^{-1}
$[KGlu]_{total}$	1.05 x 10^5 µM
D_{Glu}	220 μm^2 s^{-1}
* Gluconate binding to Ca^{2+} was approximated by a first order reaction with a dissociation constant K_D^{Glu} = 57 mM (ref. [62]); k_{on}^{Glu} was assumeed to be diffusion limited as in the case of k_{on}^{ATP}, and dissociation rate was $k_{off}^{Glu} = k_{on}^{Glu} K_D^{Glu}$	
**EGTA** (refs.[29, 56-58])	
Reaction:	
$EGTA + Ca^{2+} \underset{k_{off}^{EGTA}}{\overset{k_{on}^{EGTA}}{\rightleftarrows}} CaEGTA$	
Reaction rates:	
k_{on}^{EGTA}	10 μM^{-1} s^{-1}
k_{off}^{EGTA}	0.7 s^{-1}
$[EGTA]_{total}$	1000 µM
D_{EGTA}	220 μm^2 s^{-1}
