# Supplementary material for: Asynchronous glutamate release is enhanced in low release efficacy synapses and dispersed across the active zone
Source: Nat Commun. 2022 Jun 17;13:3497. doi: 10.1038/s41467-022-31070-4 (PMC9206079; doi:10.1038/s41467-022-31070-4)
Supplement: Supplementary file 3 — Description to Additional Supplementary Information [file 41467_2022_31070_MOESM3_ESM.pdf]

Supplementary movies legends:

Supplementary movie 1. Identification of active presynaptic boutons. The movie corresponds to the experiment presented in Fig.1. Top, raw SF-iGluSnFR fluorescence. Blinking square in the left top corner corresponds to the timing of action potentials. Middle, band-pass filtered SFiGluSnFR signal. Bottom, cumulative maximal projection of the band-pass filtered response.

Supplementary movie 2: Sub-pixel localisation of synchronous and asynchronous release events. Animated version of Fig. 5c. Scale bar 500 nm.
