# Supplementary material for: Asynchronous glutamate release is enhanced in low release efficacy synapses and dispersed across the active zone
Source: Nat Commun. 2022 Jun 17;13:3497. doi: 10.1038/s41467-022-31070-4 (PMC9206079; doi:10.1038/s41467-022-31070-4)
Supplement: Supplementary file 6 — Reporting Summary [file 41467_2022_31070_MOESM6_ESM.pdf]

nature research

Corresponding author(s): Kirill Volynski, Philipe Mendonca and Yulia Timofeeva

Last updated by author(s): Feb 6, 2022

Reporting Summary

Nature Research wishes to improve the reproducibility of the work that we publish. This form provides structure for consistency and transparency in reporting. For further information on Nature Research policies, see our Editorial Policies and the Editorial Policy Checklist.

Statistics

For all statistical analyses, confirm that the following items are present in the figure legend, table legend, main text, or Methods section.

n/a	Confirmed	
☐	☒	The exact sample size (n) for each experimental group/condition, given as a discrete number and unit of measurement
☐	☒	A statement on whether measurements were taken from distinct samples or whether the same sample was measured repeatedly
☐	☒	The statistical test(s) used AND whether they are one- or two-sided<br>*Only common tests should be described solely by name; describe more complex techniques in the Methods section.*
☒	☐	A description of all covariates tested
☐	☒	A description of any assumptions or corrections, such as tests of normality and adjustment for multiple comparisons
☐	☒	A full description of the statistical parameters including central tendency (e.g. means) or other basic estimates (e.g. regression coefficient) AND variation (e.g. standard deviation) or associated estimates of uncertainty (e.g. confidence intervals)
☐	☒	For null hypothesis testing, the test statistic (e.g. F, t, r) with confidence intervals, effect sizes, degrees of freedom and P value noted<br>*Give P values as exact values whenever suitable.*
☒	☐	For Bayesian analysis, information on the choice of priors and Markov chain Monte Carlo settings
☒	☐	For hierarchical and complex designs, identification of the appropriate level for tests and full reporting of outcomes
☒	☐	Estimates of effect sizes (e.g. Cohen's d, Pearson's r), indicating how they were calculated

Our web collection on statistics for biologists contains articles on many of the points above.

Software and code

Policy information about availability of computer code

Data collection	Electrophysiological data were acquired with WinWCP (V5.4.5, University of Strathclyde) software and Multiclamp 700B Commander software (version 2.2.0). Images were acquired with Micro-Manager software (version 1.4).
Data analysis	Image analysis was performed using ImageJ (version 152a), ImageJ ThunderSTORM plugin (version 1.3) and custom written MATLAB 2019 (MathWorks) scripts. Quantal analysis of SF-iGluSnFR responses in individual boutons and Monte Carlo simulation analysis were performed using custom written MATLAB 2019 (MathWorks). Statistical tests were performed using SigmaPlot 11 (Systat Software) and MATLAB 2019 (MathWorks) software packages. Custom MATLAB 2019 and ImageJ codes are provided with the paper within Mendonca_et_al_AnalysisScripts.zip

For manuscripts utilizing custom algorithms or software that are central to the research but not yet described in published literature, software must be made available to editors and reviewers. We strongly encourage code deposition in a community repository (e.g. GitHub). See the Nature Research guidelines for submitting code & software for further information.

Data

Policy information about availability of data

All manuscripts must include a data availability statement. This statement should provide the following information, where applicable:

- Accession codes, unique identifiers, or web links for publicly available datasets
- A list of figures that have associated raw data
- A description of any restrictions on data availability

Source experimental and modelling data are provided with this paper (SourceData.xlsx file). Raw images are available upon reasonable request from the corresponding authors.

Field-specific reporting

Please select the one below that is the best fit for your research. If you are not sure, read the appropriate sections before making your selection.

☒ Life sciences ☐ Behavioural & social sciences ☐ Ecological, evolutionary & environmental sciences

For a reference copy of the document with all sections, see nature.com/documents/nr-reporting-summary-flat.pdf

Life sciences study design

All studies must disclose on these points even when the disclosure is negative.

Sample size	The minimal numbers of boutons per recorded cell required for estimation of release efficacy and asynchronous release fraction within 80% confidence interval were evaluated using stochastic simulations of evoked vesicular release as detailed in Supplementary Fig. 9. For the number of individual cells recorded,, no statistical methods were used to pre-determine sample sizes, but our sample sizes are similar to those reported in previous publications in the field (e.g. Ermolyuk et al., Nat. Neuroscience 2013; James et al., Nat. Neuroscience 2019; Jensen et al., Nature Communications 2018).
Data exclusions	In Fig. 3a, b, boutons with at least 2 release events were selected to allow meaningful estimation of asynchronous release fraction and each recorded cell contained at least 21 boutons. Similarly, in Fig. 4, boutons with at least 2 release events were selected and each recorded cell contained at least 50 boutons. Previously Maschi and Klyachko (Neuron 2017) estimated that selection of boutons with at least 5 detected events effectively limits the localisaion analysis of vesicular release site to a sub-population of active zones that are mostly parallel to the image plane (within a 20 degree tilt). We therefore applied the same selection criteria in our analysis in Figs. 5 and 6. Furthermore, in order to compare the relative locations of synchronous and asynchronous release events (Fig. 6), only boutons that contained both event types were included.
Replication	Experimental findings were reliably reproduced among the recorded cells and individual culture preparations (minimal of 11 cells from 4 independent preparations). Main findings were reproduced independently by two researchers.
Randomization	Neuronal cultures from wild type and Syt1-/- mice were recorded in random order.
Blinding	All data analysis was performed blind to genotype.

Reporting for specific materials, systems and methods

We require information from authors about some types of materials, experimental systems and methods used in many studies. Here, indicate whether each material, system or method listed is relevant to your study. If you are not sure if a list item applies to your research, read the appropriate section before selecting a response.

Materials & experimental systems

n/a	Involved in the study
☒	☐ Antibodies
☒	☐ Eukaryotic cell lines
☒	☐ Palaeontology and archaeology
☐	☒ Animals and other organisms
☒	☐ Human research participants
☒	☐ Clinical data
☒	☐ Dual use research of concern

Methods

n/a	Involved in the study
☒	☐ ChIP-seq
☒	☐ Flow cytometry
☒	☐ MRI-based neuroimaging

Animals and other organisms

Policy information about studies involving animals; ARRIVE guidelines recommended for reporting animal research

Laboratory animals	Primary cortical neurons were produced from either wild type (C57BL/6J; Charles River) or Syt1-/- (B6; 129S-Syt1tm1Sud/J; The Jackson Laboratory) postnatal day 0 mouse pups of both sexes.
Wild animals	The study did not involve wild animals.
Field-collected samples	The study did not involve samples collected from the field.
Ethics oversight	Experiments conformed to the Animals (Scientific Procedures) Act 1986, and were approved by the UK Home Office.

Note that full information on the approval of the study protocol must also be provided in the manuscript.
