# Supplementary material for: Asynchronous glutamate release is enhanced in low release efficacy synapses and dispersed across the active zone
Source: Nat Commun. 2022 Jun 17;13:3497. doi: 10.1038/s41467-022-31070-4 (PMC9206079; doi:10.1038/s41467-022-31070-4)
Supplement: Supplementary file 8 — AnalysisScripts [file 41467_2022_31070_MOESM8_ESM.zip › Mendonca_et_al_Analysis_Scripts_Manual.pdf]

Analysis Scripts Manual

Asynchronous glutamate release is enhanced in low release efficacy synapses and dispersed across the active zone.

Authors:

Philipe R. F. Mendonça[1,2]*, Erica Tagliatti[1], Helen Langley[1], Dimitrios Kotzadimitriou[1], Criseida G. Zamora-Chimal[3], Yulia Timofeeva[1,3]* and Kirill E. Volynski[1]*

Affiliations:

[1]University College London Institute of Neurology; London, UK;

[2]Department of Physiology and Biophysics, Federal University of Minas Gerais; Brazil;

[3]Department of Computer Science, University of Warwick; Coventry, UK;

*Corresponding authors. k.volynski@ucl.ac.uk, p.mendonca@ucl.ac.uk and y.timofeeva@warwick.ac.uk

Step 1
iGluSnFR image-stack filters

a. General description:

Custom-written MATLAB script used to enhance SF-iGluSnFR visualisation of vesicular release events and to remove possible background fluorescence drift. Custom written Gaussian bandpass filter was kindly provided by Dr Hugh Robinson (University of Cambridge).

b. Instructions:

Run the self-contained *iGluSnFR_example_filter.m* script to obtain the filtered image stacks.
Input data:

- *1_Example_image_stack_original.tif*, which is an example of iGluSnFR image-stack sampled at 250Hz (we provide a section of the experiment shown in Fig. 1 of the manuscript).

Output data:

- *2_Example_image_stack_filtered.tif* bandpass filtered image-stack.
- *3_Example_image_stack_max projection.tif* maximal projection of the filtered image-stack.

Step 2
Quantal analysis of SF-iGluSnFR fluorescence traces

a. General description:

MATLAB script to define the timing and quantal amplitude of individual SF-iGluSnFR events.

b. Instructions:

- Open the self-contained *Quantal_analysis.m* script.
    - Line 16 indicates the experimental ROI selected for analysis (between 1 and 10). Setting the experimental ROI value between 1 and 10 will produce the release profile of different putative boutons specified in the corresponding ImageJ file RoiSet.zip.
    - Notes on experimental ROIs that can be selected in the provided example data set:

Exp_ROIs 1 and 8: examples of boutons with low release rate
Exp_ROIs 2, 5 and 7: examples of boutons with intermediate release rate
Exp_ROIs 3. 4 and 6: example boutons with high release rate
Exp_ROIs 9 and 10 false positive boutons. No events are detected in these ROIs
Note that Exp_ROI 6 corresponds to Bouton 2 illustrated in the manuscript Fig.1.

- Run the Quantal_Analysis.m script.

Input data:

Required data files:

- 5_ROI_data.csv. Contains the fluorescence values of all ROIs selected for analysis (putative boutons).
- 6_ephys_data.mat. Contains the electrophysiological data of the recorded cell and the frame output of the CMOS camera.

Related files from Step 1:

- 1_Example_image_stack_original.tiff. SF-iGluSnFR fluorescence image.
- 2_Example_image_stack_filtered.tiff Band passed SF-iGluSnFR fluorescence image (obtained from Step 1)
- 3_Example_image_stack_max_projection.tiff. Maximal projection of 2_Example_image_stack_filtered.tiff
- 4_RoiSet.zip. Set of ROIs to be used with ImageJ

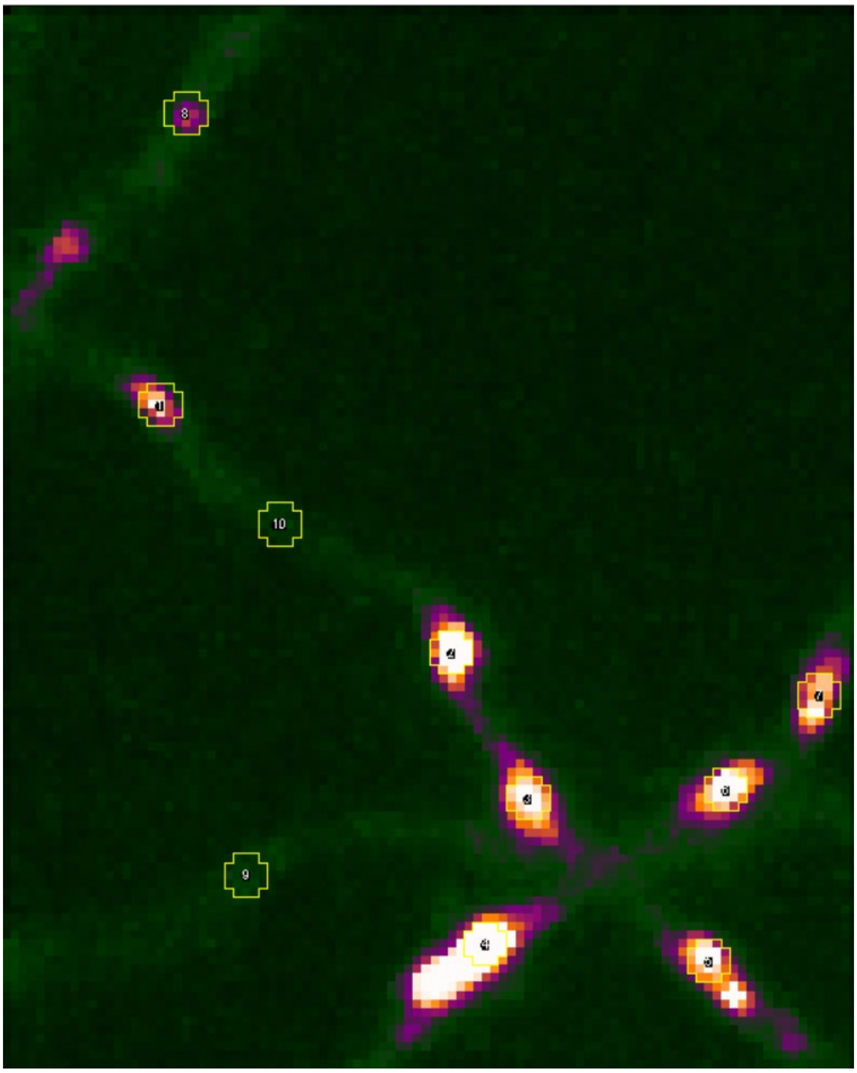

Figure 1: ROIs used for quantal analysis in the provided example dataset .

Output data:

- Three figures and one table (displayed in MATLAB command window) are generated for each bouton ROI selected for analysis:

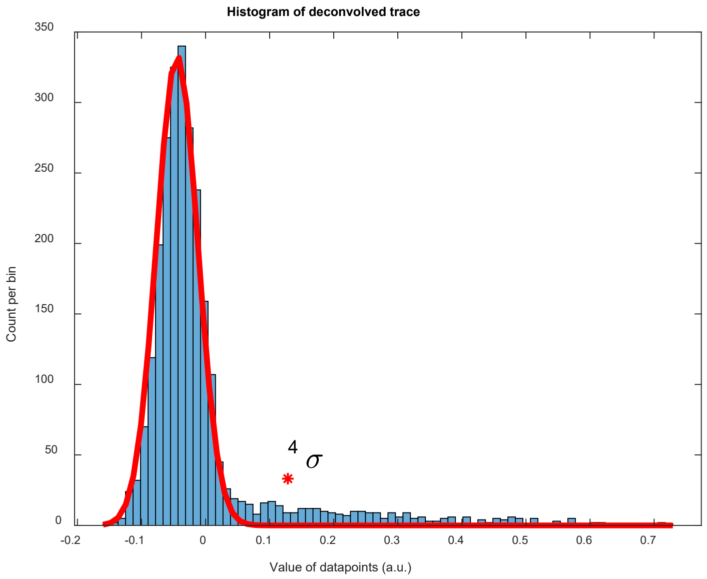

Figure 2: All-point histogram of deconvolved trace used to determine the threshold for event detection (4*SD).

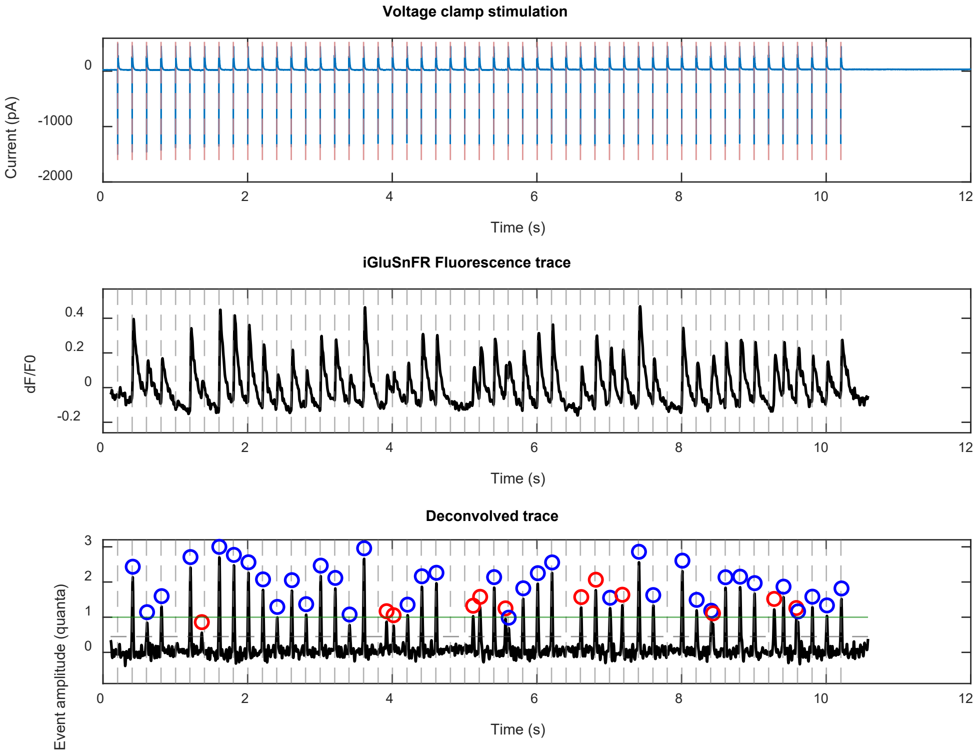

Figure 3: Quantal analysis of SF-iGluSnFR responses. Top: voltage-clamp traces showing action potentials (APs; seen as inward "escape currents"). Light red dashed lines, peak of each AP; light grey dashed, indicate the peak of each AP assigned to the nearest camera frame. Middle: Band bass-filtered SF-iGluSnFR fluorescence trace. Bottom: Deconvolved trace. Grey dashed line, event detection threshold (4*SD); green line, quantal amplitude; blue circles, synchronous events; red circles, asynchronous events

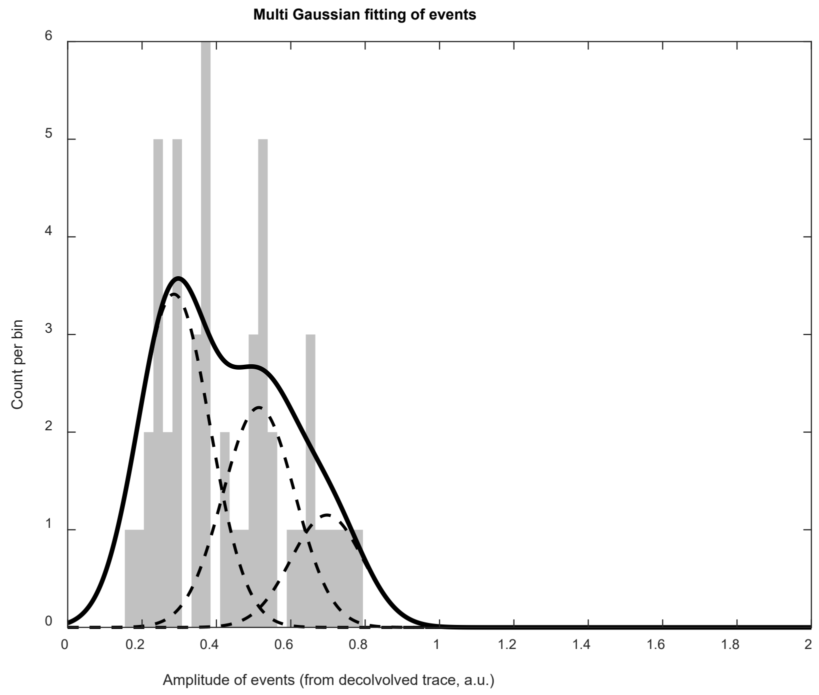

Figure 4: Multi Gaussian fitting used to estimate single quantal amplitude. Grey bars; histogram of events detected; dashed lines, individual Gaussian functions; continuous line, multi Gaussian fit.

```
Standard deviation of trace (a.u.)= 3.209881e-02
Threshold for the detection of quantal events (a.u.)= 1.283952e-01
```

Frame_of_detected_event	Quantal_amplitude_of_event	Index_of_preceding_AP	Frames_from_preceding_AP
77	2.1345	2	2
126	0.84259	3	2
176	1.2987	4	2
275	2.4093	6	2
314	0.55939	6	41
374	2.7002	8	1
424	2.4711	9	2
474	2.2609	10	2
524	1.7748	11	2
573	0.99229	12	2
623	1.7524	13	2
672	1.0704	14	2
722	2.1658	15	2
772	1.8165	16	2

Table 1: Properties of detected SF-iGluSnFR events (section printed from MATLAB command window). If SF-iGluSnFR events are detected in the selected ROI, a table is generated containing the overall features of the release: Column 1, frame where the event was detected; Column 2, quantal amplitude of the event; Column 3, Index of the action potential preceding to the analysed event and Column 4, the number of frames from the preceding action potential.

Step 3

ThunderSTORM analysis for identification of individual release sites.

a. General description

Custom-written ImageJ plugins used for sub-pixel localisation of single quanta events. contain ThunderSTORM1 and ThunderSTORM2 scripts to execute the integrated ImageJ ThunderSTORM plugin iteratively.

A summary of the procedure is illustrated in Fig.4 and Supplementary Fig. 10. The example bouton presented here is the same shown in Fig. 4.

These scripts require ImageJ 1.53f51, and the ThunderSTORM plugin needs to be installed (https://github.com/zitmen/thunderstorm/wiki/Downloads).

b1. ThunderSTORM1 Instructions:

ThunderSTORM1 estimates sub-pixel coordinates of synaptic vesicle release events.

- Open the image Noise.tif.
- Open the file ThunderSTORM1 and run it, then click Ok in the dialogue window.

Input data:

- *Noise.tif*: background noise image stack, which consists of 1,000 background images randomly sampled for each bouton (at least 15 frames away from any event). Used to determine the optimal threshold intensity value for each event.
- *All_events.tif*: event image stack, which was obtained by averaging three frames from the deconvolved image for each peak response. See Methods section, "*Sub-pixel localisation of vesicular release sites*".

Output data:

- The output of this script is a table containing the sub-pixel localisation of single quanta release events. This table is saved as *ThunderSTORM1_results.csv* file and its correspondence image is saved as *ThunderSTORM1_image.tif*.

b2. ThunderSTORM2 Instructions:

ThunderSTORM2 estimates the precision the coordinates for the vesicle release events determined in in the previous step.

- Open the image Double_noise.tif.
- Open the file ThunderSTORM2 and run it, then click Ok in the dialogue window.

Input data:

- *Double_noise.tif*: double background noise image stack, which consists of 1,000 background images randomly sampled for each bouton (at least 15 frames away from any event) and summed with another randomly sampled background image.
- *event.* A folder that contains the number of events in the current bouton example. Each event has an 'added noise' image stack (in total 50 images), which is the sum of the

original event image and a randomly selected image from the ‘background noise’ (see Methods).

Output data:

- The output of this script is a table saved as *ThunderSTORM2_results.csv* file and its correspondence image *ThunderSTORM2_image.tif* for every event with added noise.

Note: During the analysis, a folder labelled *temp_folder* is used to temporarily process the images.
