# Supplementary figures and images for: Asynchronous glutamate release is enhanced in low release efficacy synapses and dispersed across the active zone
Source: Nat Commun. 2022 Jun 17;13:3497. doi: 10.1038/s41467-022-31070-4 (PMC9206079; doi:10.1038/s41467-022-31070-4)

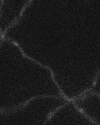

Supplement: Supplementary file 8 — AnalysisScripts [file 41467_2022_31070_MOESM8_ESM.zip › Step 1 Image filter/1_Example_image_stack_original.tif]

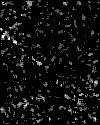

Supplement: Supplementary file 8 — AnalysisScripts [file 41467_2022_31070_MOESM8_ESM.zip › Step 1 Image filter/2_Example_image_stack_filtered.tif]

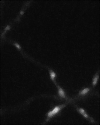

Supplement: Supplementary file 8 — AnalysisScripts [file 41467_2022_31070_MOESM8_ESM.zip › Step 1 Image filter/3_Example_image_stack_max projection.tif]

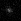

Supplement: Supplementary file 8 — AnalysisScripts [file 41467_2022_31070_MOESM8_ESM.zip › Step 3 ThunderStorm/All_events.tif]

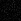

Supplement: Supplementary file 8 — AnalysisScripts [file 41467_2022_31070_MOESM8_ESM.zip › Step 3 ThunderStorm/Double_noise.tif]

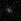

Supplement: Supplementary file 8 — AnalysisScripts [file 41467_2022_31070_MOESM8_ESM.zip › Step 3 ThunderStorm/event/e_1.tif]

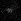

Supplement: Supplementary file 8 — AnalysisScripts [file 41467_2022_31070_MOESM8_ESM.zip › Step 3 ThunderStorm/event/e_10.tif]

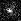

Supplement: Supplementary file 8 — AnalysisScripts [file 41467_2022_31070_MOESM8_ESM.zip › Step 3 ThunderStorm/event/e_11.tif]

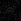

Supplement: Supplementary file 8 — AnalysisScripts [file 41467_2022_31070_MOESM8_ESM.zip › Step 3 ThunderStorm/event/e_12.tif]

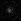

Supplement: Supplementary file 8 — AnalysisScripts [file 41467_2022_31070_MOESM8_ESM.zip › Step 3 ThunderStorm/event/e_13.tif]

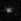

Supplement: Supplementary file 8 — AnalysisScripts [file 41467_2022_31070_MOESM8_ESM.zip › Step 3 ThunderStorm/event/e_14.tif]

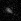

Supplement: Supplementary file 8 — AnalysisScripts [file 41467_2022_31070_MOESM8_ESM.zip › Step 3 ThunderStorm/event/e_15.tif]

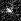

Supplement: Supplementary file 8 — AnalysisScripts [file 41467_2022_31070_MOESM8_ESM.zip › Step 3 ThunderStorm/event/e_16.tif]

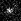

Supplement: Supplementary file 8 — AnalysisScripts [file 41467_2022_31070_MOESM8_ESM.zip › Step 3 ThunderStorm/event/e_17.tif]

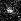

Supplement: Supplementary file 8 — AnalysisScripts [file 41467_2022_31070_MOESM8_ESM.zip › Step 3 ThunderStorm/event/e_18.tif]

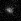

Supplement: Supplementary file 8 — AnalysisScripts [file 41467_2022_31070_MOESM8_ESM.zip › Step 3 ThunderStorm/event/e_19.tif]

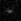

Supplement: Supplementary file 8 — AnalysisScripts [file 41467_2022_31070_MOESM8_ESM.zip › Step 3 ThunderStorm/event/e_2.tif]

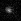

Supplement: Supplementary file 8 — AnalysisScripts [file 41467_2022_31070_MOESM8_ESM.zip › Step 3 ThunderStorm/event/e_20.tif]

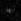

Supplement: Supplementary file 8 — AnalysisScripts [file 41467_2022_31070_MOESM8_ESM.zip › Step 3 ThunderStorm/event/e_21.tif]

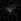

Supplement: Supplementary file 8 — AnalysisScripts [file 41467_2022_31070_MOESM8_ESM.zip › Step 3 ThunderStorm/event/e_22.tif]

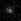

Supplement: Supplementary file 8 — AnalysisScripts [file 41467_2022_31070_MOESM8_ESM.zip › Step 3 ThunderStorm/event/e_23.tif]

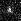

Supplement: Supplementary file 8 — AnalysisScripts [file 41467_2022_31070_MOESM8_ESM.zip › Step 3 ThunderStorm/event/e_24.tif]

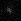

Supplement: Supplementary file 8 — AnalysisScripts [file 41467_2022_31070_MOESM8_ESM.zip › Step 3 ThunderStorm/event/e_25.tif]

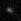

Supplement: Supplementary file 8 — AnalysisScripts [file 41467_2022_31070_MOESM8_ESM.zip › Step 3 ThunderStorm/event/e_26.tif]

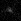

Supplement: Supplementary file 8 — AnalysisScripts [file 41467_2022_31070_MOESM8_ESM.zip › Step 3 ThunderStorm/event/e_27.tif]

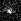

Supplement: Supplementary file 8 — AnalysisScripts [file 41467_2022_31070_MOESM8_ESM.zip › Step 3 ThunderStorm/event/e_28.tif]

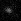

Supplement: Supplementary file 8 — AnalysisScripts [file 41467_2022_31070_MOESM8_ESM.zip › Step 3 ThunderStorm/event/e_29.tif]

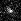

Supplement: Supplementary file 8 — AnalysisScripts [file 41467_2022_31070_MOESM8_ESM.zip › Step 3 ThunderStorm/event/e_3.tif]

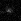

Supplement: Supplementary file 8 — AnalysisScripts [file 41467_2022_31070_MOESM8_ESM.zip › Step 3 ThunderStorm/event/e_30.tif]

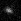

Supplement: Supplementary file 8 — AnalysisScripts [file 41467_2022_31070_MOESM8_ESM.zip › Step 3 ThunderStorm/event/e_31.tif]

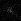

Supplement: Supplementary file 8 — AnalysisScripts [file 41467_2022_31070_MOESM8_ESM.zip › Step 3 ThunderStorm/event/e_32.tif]

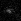

Supplement: Supplementary file 8 — AnalysisScripts [file 41467_2022_31070_MOESM8_ESM.zip › Step 3 ThunderStorm/event/e_33.tif]

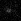

Supplement: Supplementary file 8 — AnalysisScripts [file 41467_2022_31070_MOESM8_ESM.zip › Step 3 ThunderStorm/event/e_34.tif]

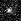

Supplement: Supplementary file 8 — AnalysisScripts [file 41467_2022_31070_MOESM8_ESM.zip › Step 3 ThunderStorm/event/e_35.tif]

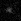

Supplement: Supplementary file 8 — AnalysisScripts [file 41467_2022_31070_MOESM8_ESM.zip › Step 3 ThunderStorm/event/e_36.tif]

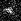

Supplement: Supplementary file 8 — AnalysisScripts [file 41467_2022_31070_MOESM8_ESM.zip › Step 3 ThunderStorm/event/e_37.tif]

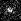

Supplement: Supplementary file 8 — AnalysisScripts [file 41467_2022_31070_MOESM8_ESM.zip › Step 3 ThunderStorm/event/e_4.tif]

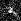

Supplement: Supplementary file 8 — AnalysisScripts [file 41467_2022_31070_MOESM8_ESM.zip › Step 3 ThunderStorm/event/e_5.tif]

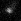

Supplement: Supplementary file 8 — AnalysisScripts [file 41467_2022_31070_MOESM8_ESM.zip › Step 3 ThunderStorm/event/e_6.tif]

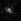

Supplement: Supplementary file 8 — AnalysisScripts [file 41467_2022_31070_MOESM8_ESM.zip › Step 3 ThunderStorm/event/e_7.tif]

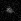

Supplement: Supplementary file 8 — AnalysisScripts [file 41467_2022_31070_MOESM8_ESM.zip › Step 3 ThunderStorm/event/e_8.tif]

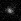

Supplement: Supplementary file 8 — AnalysisScripts [file 41467_2022_31070_MOESM8_ESM.zip › Step 3 ThunderStorm/event/e_9.tif]

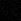

Supplement: Supplementary file 8 — AnalysisScripts [file 41467_2022_31070_MOESM8_ESM.zip › Step 3 ThunderStorm/Noise.tif]
